# Cost Effectiveness of Two Short Implants Versus One Short Implant With a Cantilever in the Posterior Region: 7.5‐Year Follow‐Up of a Randomised Controlled Trial

**DOI:** 10.1111/jcpe.70039

**Published:** 2025-09-21

**Authors:** Franz J. Strauss, Lucia Schiavon, Nadja Naenni, Riccardo D. Kraus, Gustavo Sáenz‐Ravello, Nicolas Müller, Ronald E. Jung, Daniel S. Thoma

**Affiliations:** ^1^ Clinic of Reconstructive Dentistry University of Zurich Zurich Switzerland; ^2^ Universidad Autonoma de Chile Santiago Chile; ^3^ Department of Neurosciences, School of Dentistry University of Padua Padua Italy; ^4^ Center for Surveillance and Epidemiology of Oral Diseases, Faculty of Dentistry University of Chile Santiago Chile; ^5^ Department of Periodontology, Research Institute for Periodontal Regeneration Yonsei University College of Dentistry Seoul Republic of Korea

## Abstract

**Aim:**

To compare long‐term clinical and radiographic outcomes of a single short implant (6 mm) supporting a cantilevered restoration versus two adjacent short implants with non‐splinted single crowns over a 7.5‐year follow‐up and determine which approach is more cost effective.

**Materials and Methods:**

A total of 36 patients with at least a two‐tooth gap in the posterior region were randomised to receive either one short implant with a cantilever prosthesis (ONE‐C) or two short implants with individual crowns (TWO). Fixed restorations were placed 3–6 months post implantation, and patients were evaluated at baseline and at 6 months and 1, 3, 5 and 7.5 years. Kaplan–Meier curves, mixed‐effects models and cost‐effectiveness models were used to compare the groups.

**Results:**

Twenty‐five patients (15 in ONE‐C, 10 in TWO) completed the 7.5‐year follow‐up. Implant survival was 83.3% in group ONE‐C and 86.6% in group TWO, with no significant differences between the groups (*p* = 0.772). No statistically significant differences were found between groups for marginal bone levels (mean difference −0.16 [95% CI: −0.7 to 0.3] *p* = 0.57), probing depth (mean difference −0.13 [95% CI: −0.5 to 0.3] *p* = 0.58), bleeding on probing (mean difference 0.0 [95% CI: −0.0; 0.2] *p* = 0.31) or plaque levels (mean difference −0.0 [95% CI: −0.1 to 0.1] *p* = 0.93). Technical complications were more frequent in the ONE‐C group (64%) than in the TWO group (36%).

**Conclusion:**

Both treatment approaches showed comparable clinical and radiographic outcomes. Short implants supporting cantilever restorations were generally more cost effective than two short implants but exhibited higher early complication and failure rates, likely related to mechanical overload.

**Trial Registration:**

ClinicalTrials.gov Identifier: NCT01649531

## 1 Introduction

After tooth loss, physiological bone resorption often complicates traditional implant placement, particularly in the posterior regions where the ridge height may be limited by anatomical structures such as the maxillary sinus or inferior alveolar nerve (Sculean et al. 2019). This issue is even more pronounced in patients with periodontitis, where tooth loss can occur from the progressive breakdown of the tooth‐supporting structures, including the alveolar bone (Sanz et al. 2020). In these cases, placement of standard‐length implants might require extensive and invasive surgical procedures such as primary bone augmentation, guided bone regeneration (GBR) and lateral or crestal sinus lift (Sáenz‐Ravello et al. 2023). However, these augmentative procedures present considerable risk of complications, ranging from 6.7% to 44% for sinus floor elevation (Danesh‐Sani et al. 2016; Schiavon et al. 2022) and 3.5%–12.9% for GBR (Urban et al. 2019). To minimise these risks, short dental implants (e.g., < 8 mm) have been introduced as a less invasive alternative (Shi et al. 2021; Thoma, Haas, et al. 2015). They help avoid complex and invasive surgeries, reduce treatment time and costs and improve patient outcomes (Shi et al. 2021). Systematic reviews have confirmed their efficacy, reporting survival rates of up to 99% over an 18‐month follow‐up, marginal bone loss values comparable to longer implants with bone augmentation and reduced postoperative morbidity (Esposito et al. 2011; Sáenz‐Ravello et al. 2023; Thoma, Zeltner, et al. 2015).

In posterior jaws, a common clinical scenario is the presence of a two‐tooth edentulous space. The standard treatment is the placement of two adjacent single implants. However, this is not always possible in cases with a limited mesio‐distal space or pre‐existing bone defects. An alternative is the placement of a single implant supporting a cantilever prosthesis. This approach extends the prosthesis beyond the supporting implant to replace the missing tooth without the need for additional implants. This option reduces the treatment costs (Thoma et al. 2021), shortens treatment time and lowers surgical invasiveness while still providing a functional restoration (Roccuzzo, Fanti, et al. 2023; Thoma et al. 2021). Cantilever designs, nevertheless, have raised concerns due to the potential for increased occlusal and functional stresses. These stresses could lead to biological complications such as increased radiographic bone density patterns (Gil et al. 2022). However, several clinical studies have shown no significant difference in marginal bone loss between cantilever and non‐cantilever restorations (Aglietta et al. 2012; Palmer et al. 2012; Roccuzzo et al. 2020; Romeo et al. 2003; Romeo et al. 2009; Schmid et al. 2020; Schmid et al. 2021; Wennstrom et al. 2004). While these findings are promising, they should be interpreted with caution because most studies are retrospective and lack control groups.

Both short implants and single implants supporting cantilever restorations have proven to be reliable options in the posterior atrophic mandible. When combined, these approaches might further expand treatment options by reducing surgical invasiveness, patient morbidity and treatment time (Schmid et al. 2020; Thoma et al. 2021). However, cost effectiveness remains uncertain, as no study has directly compared the two interventions, and no RCTs with long‐term follow‐up are available. To address this gap, in the present RCT we compared the clinical, radiographic and technical outcomes of a single short implant supporting a cantilever restoration to two adjacent short implants with non‐splinted single crowns, along with their relative cost effectiveness over a 7.5‐year follow‐up.

## 2 Materials and Methods

This is the 7.5‐year follow‐up study of a previously published RCT (Thoma et al. 2021) and is reported in accordance with the CONSORT guidelines for parallel‐group randomised trials (Moher et al. 2010).

### 2.1 Study Design

This study was designed as an RCT with two parallel groups, spanning a period of 10 years and conducted at the Clinic of Reconstructive Dentistry, Center of Dental Medicine, University of Zurich, Switzerland. The study protocol was approved by the local ethics committee (KEK‐ZH‐Nr 2012–0097) and registered at ClinicalTrials.gov (NCT01649531).

### 2.2 Study Population

Thirty‐six patients requiring an implant‐supported fixed dental prosthesis (FDP) and meeting the following inclusion criteria were consecutively enrolled:

- Age ≥ 18 years.
- Healthy individuals classified as ASA I or II according to the American Society of Anaesthesiology (https://doi.org/10.1097/01.ASM.0001073116.40041.ee), aged ≥ 18 years.
- No systemic medical conditions contraindicating implant therapy (Bornstein et al. 2009).
- Presence of two adjacent missing teeth in the maxilla or mandible, from the first premolar to the second molar.
- At least one natural tooth adjacent to the edentulous space.
- Sufficient vertical bone height:
  ○ ≥ 8 mm in the mandible (to allow placement of a 6‐mm implant with a 2‐mm safety margin from the inferior alveolar nerve).
  ○ ≥ 6 mm in the maxilla (from the alveolar crest to the sinus floor).
- Absence of periodontal disease.
- Good oral hygiene, defined as a full‐mouth plaque index < 25% (O'Leary et al. 1972).
- Adequate control of inflammation, defined as full‐mouth bleeding on probing (BOP) < 25% (Ainamo and Bay 1975).

### 2.3 Randomisation

Participants were randomly assigned to receive either a single short implant (ONE‐C) or two short implants (TWO) based on a computer‐generated randomisation list. All implants were 6 mm in length and 4.1 mm in diameter (Straumann Standard Plus, SLActive; Straumann AG, Basel, Switzerland). Allocation concealment was maintained using sealed envelopes, opened only after flap elevation.

### 2.4 Implant Surgery

Following the elevation of a full‐thickness flap, implant sites were prepared following the manufacturer's recommendations. In the ONE‐C group, a single implant was placed in the site with the most favourable bone conditions (based on vertical and horizontal bone availability), generally favouring the distal site to allow for a mesial cantilever. In the TWO group, two implants were placed. When minor bone dehiscence was identified after implant placement, GBR was performed using demineralised bovine bone mineral (Bio‐Oss; Geistlich AG, Wolhusen, Switzerland) covered with a resorbable collagen membrane (Bio‐Gide; Geistlich AG). After periosteal release, the flap was repositioned and sutured. Both approaches, namely submerged and transmucosal healing, were allowed under the study protocol.

### 2.5 Prosthetic Procedure

**FIGURE 1:**
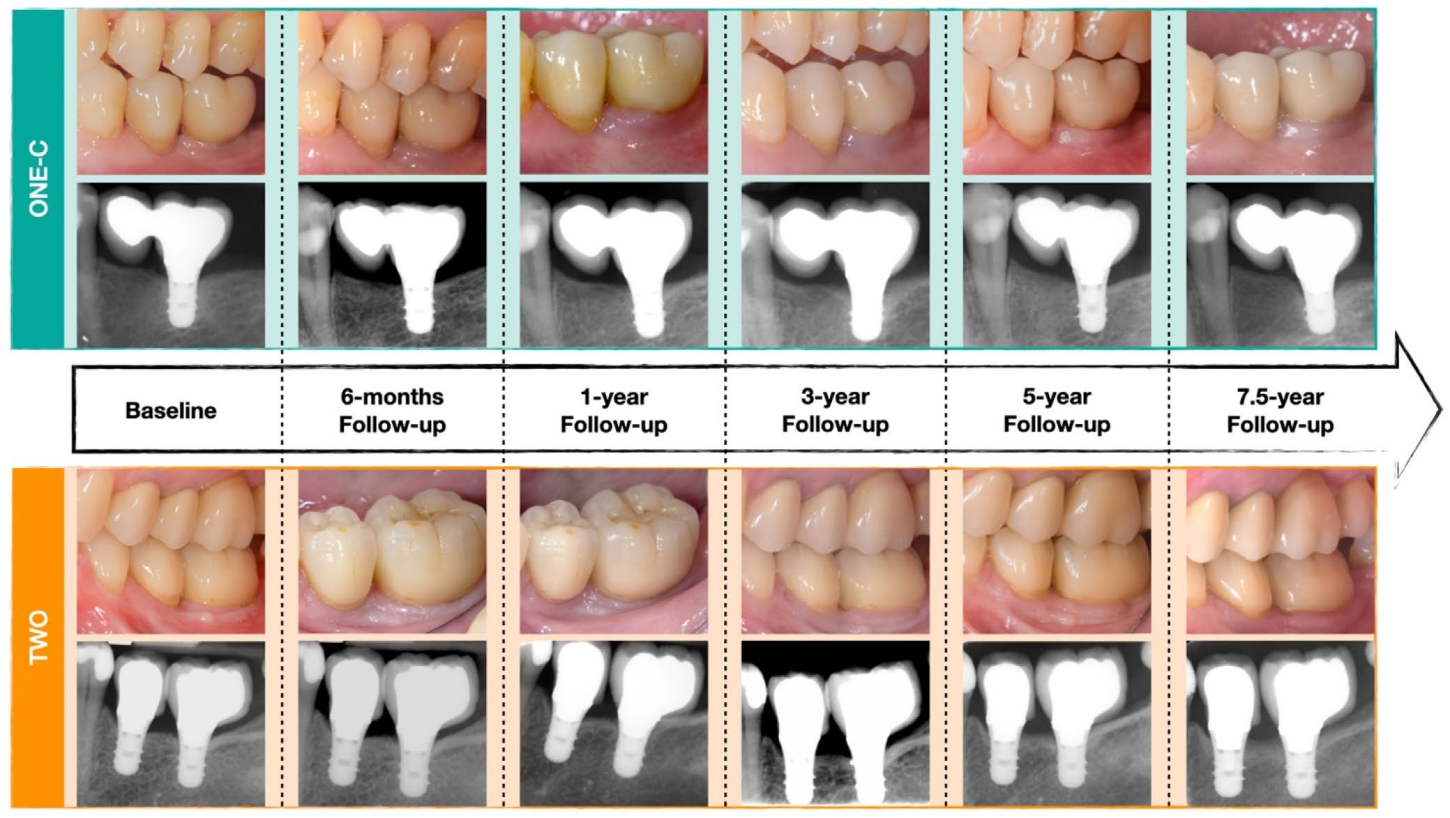
Clinical and radiographic images of one representative case for each group during the 7.5‐year follow‐up period.

All prosthetic procedures followed the manufacturer's recommendations. All implants were restored using a delayed loading protocol (3–6 months). Group ONE‐C received a single crown with a cantilever, while group TWO received two non‐splinted single crowns (Figure 1). All the implant‐supported FDPs were screw‐retained, porcelain‐fused‐to‐metal (PFM) restorations with occlusion designed to follow group function. Baseline examination was performed 1–3 weeks after prosthesis delivery. Follow‐up assessments were then performed at 6 months, 1 year, 3 years, 5 and 7.5 years post loading. Each participant was enrolled in a customised maintenance programme, including regular dental hygiene sessions every 3–12 months.

### 2.6 Study Outcomes

#### 2.6.1 Implant and Reconstruction Survival

Implant survival was defined as the implant remaining in place and stable on manual testing. Reconstruction survival was defined as the FDPs remaining in place. Both implant and reconstruction survival were measured at the patient level at the 7.5‐year follow‐up timepoint.

#### 2.6.2 Biological and Technical Complications

Biological complications, including peri‐implant mucositis and peri‐implantitis, were diagnosed according to the 2017 World Workshop on the Classification of Periodontal and Peri‐Implant Diseases and Conditions (Berglundh et al. 2018; Renvert et al. 2018).

Peri‐implant mucositis case definition (Renvert et al. 2018) was as follows:

- Presence of profuse bleeding (line or drop) and/or suppuration on probing.
- Increased probing depth (PD) compared to baseline,
- Absence of bone loss beyond initial crestal bone–level changes due to normal remodelling.

Peri‐implantitis case definition (Berglundh et al. 2018; Renvert et al. 2018) was as follows:

- Presence of bleeding and/or suppuration on gentle probing.
- Increased PD compared to previous examinations.
- Bone loss beyond crestal bone–level changes (≥ 2 mm from baseline).
- PD ≥ 6 mm.

Technical complications included implant fracture, abutment fracture, ceramic chipping, screw loosening and screw fracture. These complications were managed with appropriate clinical interventions.

#### 2.6.3 Radiographic Outcomes (Primary Outcome)

Standardised intraoral radiographs were taken using a paralleling technique with Rinn holders and digital films at baseline and up to 7.5 years after loading. The pitch distance between two implant threads was used to calibrate the images and determine the exact magnification. Marginal bone levels (MBLs) were measured from the implant shoulder to the bone crest on both the mesial and distal sites, and the average of the two was calculated for each implant.

#### 2.6.4 Clinical Parameters

At each follow‐up visit, the following clinical parameters were recorded at six sites per implant and averaged:

- PD.
- BOP percentage (%) (Ainamo and Bay 1975).
- Plaque control record (PCR) percentage (%) (O'Leary et al. 1972).

### 2.7 Sample Size Calculation

Sample size calculation was done on the basis of the expected differences in the primary outcome (radiographic MBL) between the two treatment groups. Power analysis was conducted using a two‐sample *t*‐test, taking as reference data from previous studies (Albrektsson et al. 1986; Palmer et al. 1997). Assuming a 0.5 mm difference in the primary outcome between the groups and a common standard deviation of 0.46 mm, the two‐tailed effect size for the *t*‐test was calculated to be 1.086. With a 5% type‐I error rate, 80% power and a 15% drop‐out rate, 18 participants per group were required to detect a 0.5 mm difference in the primary outcome between the treatment arms.

### 2.8 Statistical Analysis

Descriptive statistics were used for all parameters: means, standard deviations (SD), and medians were reported for continuous variables, while frequencies were used for categorical variables. Changes in clinical and radiographic outcomes, both within and between treatment groups, were analysed using linear mixed‐effects models, which accounted for within‐subject correlations due to repeated measurements. Fixed effects included treatment group, time and their interaction, enabling the estimation of treatment effects at each timepoint. Patients were treated as random effects. Group differences were assessed using the linear contrast command. Model assumptions were evaluated visually using residual diagnostics, including *Q*–*Q* plots and histograms. When assumptions were not met, nonparametric general linear models with generalised estimating equations (GEEs) were applied. Kaplan–Meier analysis was used to estimate cumulative patient‐level survival probabilities, and group comparisons were conducted using the log‐rank test. A two‐sided significance level of 5% (α = 0.05) was applied throughout. All statistical analyses were performed using Stata v18.0 (StataCorp, College Station, TX, USA).

### 2.9 Cost‐Effectiveness Analysis

The health economic evaluation adhered to the CHEERS 2022 guidelines and was conducted over a 7.5‐year horizon from the Swiss private‐payer perspective. A three‐state Markov microsimulation (functional, complication, failure) with 6‐month cycles and 3% annual discounting modelled the outcomes based on trial‐derived hazards. Effectiveness was expressed in implant‐years, with temporary decrements for complication episodes. Costs included initial rehabilitation (ONE‐C: CHF 5755; TWO: CHF 6465) and complication management, all valued in 2025 Swiss francs (CHF). Incremental cost‐effectiveness ratios and net monetary benefit were calculated, with uncertainty explored through deterministic sensitivity analyses and 20,000‐iteration probabilistic simulations, summarised in cost‐effectiveness planes and acceptability curves. Scenario analyses and cost‐minimisation analysis were also performed. Model validity was assessed by expert review, internal consistency checks and comparison with observed trial outcomes (see Data S1).

## 3 Results

### 3.1 Demographics and Main Characteristics

A total of 36 patients (mean age: 67.5 ± 11.6 years) were enrolled and received 54 implants: 18 in group ONE‐C and 36 in group TWO. Patient demographics are presented in Table 1 and the implant distribution and location are presented in Figure S1.

**TABLE 1:** Baseline characteristics of the study subjects.

	Group ONE‐C	Group TWO	Overall
Patients			
*N* (%)	19 (54.3%)	16 (45.7%)	35 (100%)
Age			
Years, mean (SD)	65.3 (7.6)	55.2 (12.7)	60.7 (11.3)
Gender			
Female (%)	14 (73.7%)	11 (68.8%)	25 (71.4%)
Male (%)	5 (26.3%)	5 (31.3%)	5 (28.6%)
Implant position			
14 (%)	0 (0.00%)	3 (9.4%)	3 (5.9%)
15 (%)	2 (10.5%)	4 (12.5%)	6 (11.8%)
16 (%)	0 (0.0%)	1 (3.1%)	1 (2.0%)
17 (%)	0 (0.0%)	0 (3.1%)	1 (2.0%)
24 (%)	0 (0.0%)	0 (3.1%)	1 (2.0%)
25 (%)	1 (5.3%)	0 (0.0%)	1 (2.0%)
26 (%)	3 (15.8%)	0 (0.0%)	3 (5.9%)
34 (%)	0 (0.0%)	1 (3.1%)	1 (2.0%)
35 (%)	0 (0.0%)	8 (25.0%)	8 (15.7%)
36 (%)	4 (21.1%)	7 (21.9%)	11 (21.6%)
37 (%)	2 (10.5%)	0 (0.0%)	2 (3.9%)
44 (%)	1 (5.3%)	1 (3.1%)	2 (3.9%)
45 (%)	1 (5.3%)	2 (6.3%)	3 (5.9%)
46 (%)	5 (26.3%)	2 (6.3%)	7 (13.7%)
47 (%)	0 (0.0%)	1 (3.1%)	1 (2.0%)

**FIGURE 2:**
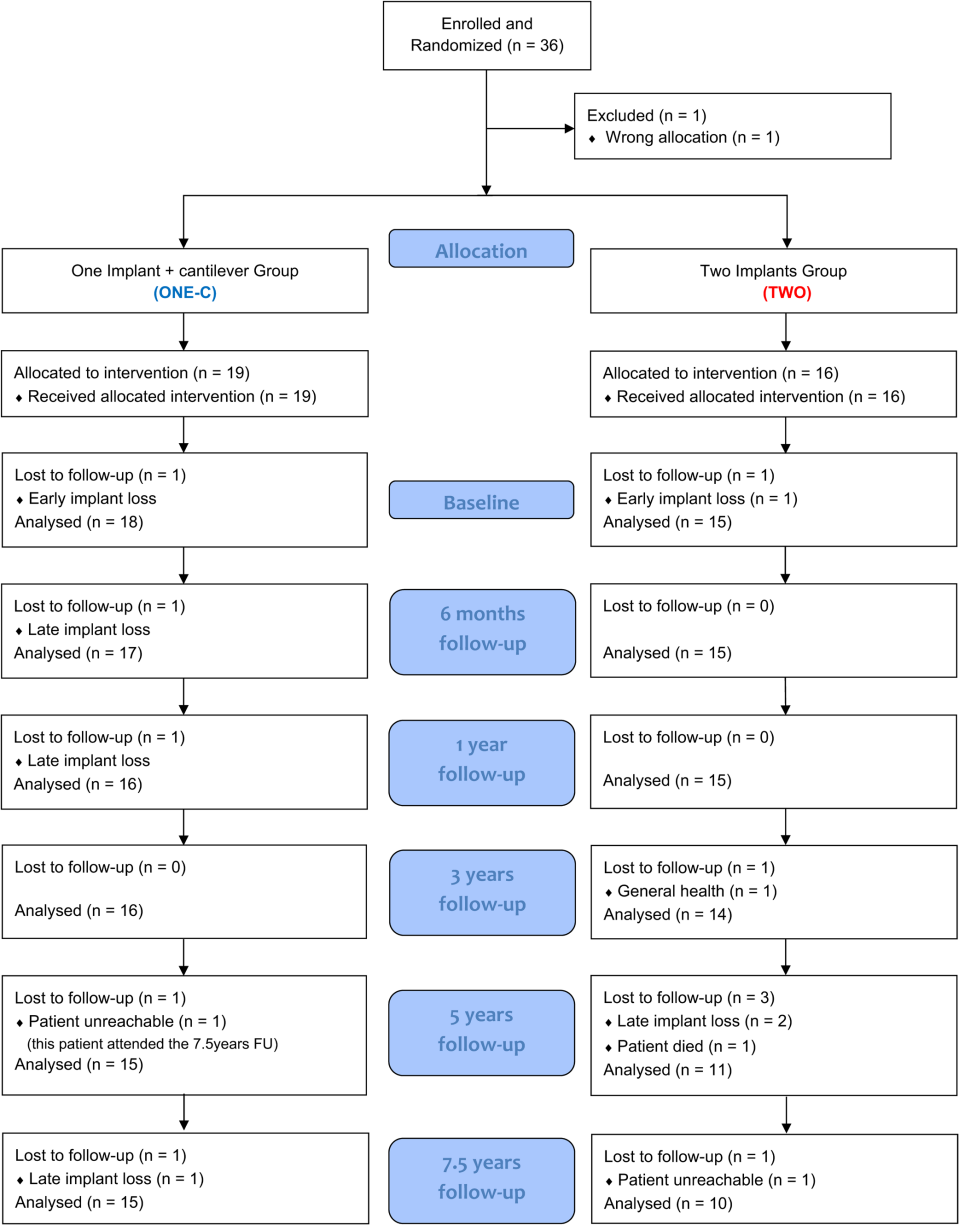
Study flow diagram.

**FIGURE 3:**
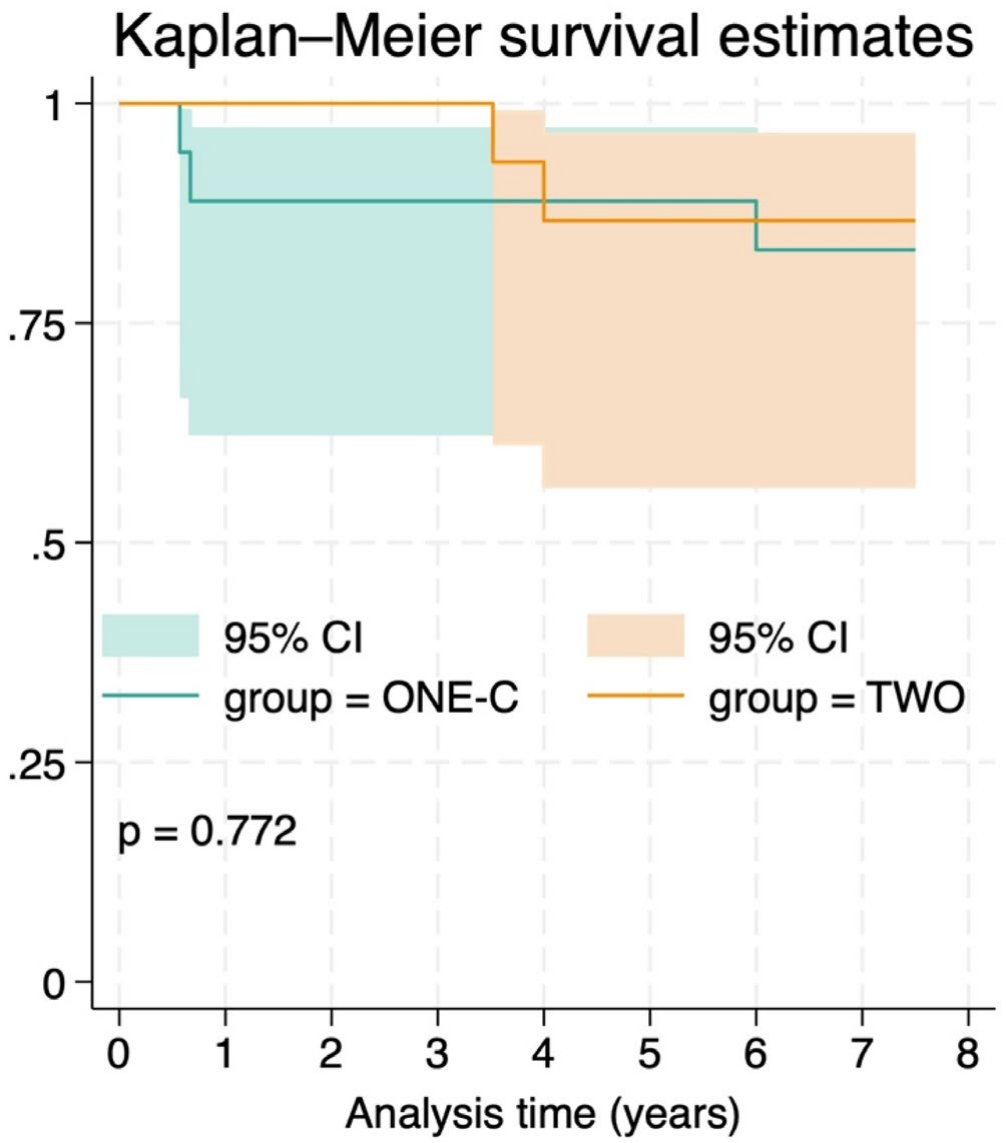
Kaplan–Meier estimates of survival in both treatment groups up to 7.5‐year follow‐up. No significant differences were found between the groups (log‐rank test, *p* = 0.772). Shaded areas represent 95% confidence intervals.

During the follow‐up, three patients from group TWO were lost to follow‐up (non‐response or death), and five late implant failures were recorded (Figure 2). At the 7.5‐year follow‐up, 25 patients (15 in group ONE‐C; 10 in group TWO) attended the examination visit. During the 7.5‐year follow‐up, five late implant failures occurred (three in group ONE‐C group and 2 in group TWO). All implant failures occurred in the mandible. In group TWO, both failures involved distal implant sites. At the patient level, the 7.5‐year implant survival rate was 83.3% (CI: 56.7–94.3) in group ONE‐C and 86.6% (CI: 56.3–96.5) in group TWO (Kaplan–Mayer; Figure 3) with no significant differences between the groups (*p* = 0.772).

### 3.2 Technical Complications

Over the 7.5‐year follow‐up period, 44 technical complications occurred in 18 patients: 28 complications in group ONE‐C and 16 in group TWO. The most common complication was ceramic chipping (57%), followed by screw loosening (23%). At all timepoints, the incidence of technical complications tended to be higher in the group ONE‐C than in the group TWO (Table 2).

**TABLE 2:** Type, number and percentage of complications occurred in the two study groups at the investigated timepoints.

Complications	6‐month FU	1‐year FU	3‐year FU	5‐year FU	7.5‐year FU	Overall		Group
1. Screw loosening	2	1	2	3	0	8	18%	Group ONE‐C
2. Screw fracture	0	0	0	0	0	0		
3. Fracture of veneering ceramic	2	2	3	5	3	15	34%	
4. Framework fracture	0	0	0	0	0	0		
5. Implant fracture	0	0	0	0	0	0		
6. Other	1	0	2	1	1	5	11%	
Total complication per timepoint	5	3	7	9	4	28	64%	
1. Screw loosening	0	0	0	2	0	2	5%	Group TWO
2. Screw fracture	0	0	0	0	0	0		
3. Fracture of veneering ceramic	1	2	2	2	3	10	23%	
4. Framework fracture	0	0	0	0	0	0		
5. Implant fracture	0	0	0	0	0	0		
6. Other	1	0	1	0	2	4	9%	
Total complications per timepoint	2	2	3	4	5	16	36%	
1. Screw loosening	2	1	2	5	0	10	23%	Overall
2. Screw fracture	0	0	0	0	0	0		
3. Fracture of veneering ceramic	3	4	5	7	6	25	57%	
4. Framework fracture	0	0	0	0	0	0		
5. Implant fracture	0	0	0	0	0	0		
6. Other	2	0	3	1	3	9	20%	
Total complication per timepoint	7	5	10	13	9	44	100%	

Abbreviation: FU, follow‐up.

### 3.3 Clinical Outcome Measures

Clinical parameters at the patient level are summarised in Table 3. No statistically significant differences were found between groups for plaque levels (mean difference −0.0 [95% CI: −0.1 to 0.1] *p* = 0.93), bleeding on probing (mean difference 0.0 [95% CI: −0.0 to 0.2] *p* = 0.31) or probing depth (mean difference −0.13 [95% CI: −0.5 to 0.3] *p* = 0.58) at any timepoint. PD increased significantly compared to baseline at the 3‐year follow‐up (*p* = 0.04), while no further significant changes were observed thereafter. PCR increased significantly after baseline but remained stable during subsequent follow‐up. BOP increased significantly at 5 and 7.5 years (*p* < 0.05 for both). At 7.5 years, the prevalence of peri‐implant mucositis was 47% in group ONE‐C and 67% in group TWO. The prevalence of peri‐implantitis was 7% (one case) in group ONE‐C; no cases were found in group TWO.

**TABLE 3:** Clinical and radiographic outcomes for the two study groups at the investigated time points.

Parameter	Timepoint	Group ONE‐C		Group TWO		Comparison	
		Mean (SD)	Median (Q1; Q3)	Mean (SD)	Median (Q1; Q3)	Adjusted mean treatment difference (95% CI)	*p*‐value
Plaque control record	Baseline	0.01 (0.03)	0.00 (0.00; 0.00)	0.02 (0.04)	0.00 (0.00; 0.04)	0.01 (−0.01; 0.04)	0.26
	6‐month FU	0.11 (0.18)	0.00 (0.00; 0.17)	0.17 (0.27)	0.08 (0.00; 0.17)	0.07 (−0.09; 0.23)	0.39
	1‐year FU	0.11 (0.26)	0.00 (0.00; 0.17)	0.21 (0.29)	0.08 (0.00; 0.29)	0.09 (−0.10; 0.28)	0.36
	3‐year FU	0.12 (0.14)	0.08 (0.00; 0.19)	0.21 (0.22)	0.21 (0.02; 0.31)	0.09 (−0.05; 0.22)	0.20
	5‐year FU	0.18 (0.17)	0.17 (0.04; 0.29)	0.26 (0.15)	0.33 (0.17; 0.33)	0.05 (−0.07; 0.17)	0.40
	7.5‐year FU	0.12 (0.12)	0.17 (0.00; 0.17)	0.13 (0.16)	0.08 (0.00; 0.17)	−0.01 (−0.12; 0.12)	0.93
Probing depth	Baseline	2.88 (0.80)	2.75 (2.50; 3.33)	2.89 (0.37)	3.00 (2.67; 3.13)	0.01 (−0.39; 0.42)	0.96
	6‐month FU	2.77 (0.75)	2.67 (2.17; 3.17)	2.81 (0.51)	2.67 (2.42; 3.33)	0.03 (−0.38; 0.44)	0.89
	1‐year FU	2.86 (0.57)	2.83 (2.29; 3.33)	2.87 (0.44)	2.83 (2.58; 3.21)	−0.01 (−0.42; 0.41)	0.97
	3‐year FU	3.29 (0.54)	3.25 (2.96; 3.71)	3.20 (0.60)	3.20 (2.94; 3.40)	−0.12 (−0.54; 0.30)	0.58
	5‐year FU	3.09 (0.92)	3.00 (2.67; 3.67)	2.98 (0.39)	3.00 (2.67; 3.25)	−0.13 (−0.57; 0.31)	0.56
	7‐year FU	3.11 (0.74)	3.00 (2.97; 3.67)	3.02 (0.49)	3.04 (2.75; 3.33)	−0.13 (−0.58; 0.32)	0.58
Beeding on probing	Baseline	0.19 (0.19)	0.17 (0.00; 0.33)	0.12 (0.13)	0.08 (0.00; 0.17)	−0.08 (0.23; 0.78)	0.32
	6‐month FU	0.22 (0.17)	0.17 (0.00; 0.33)	0.22 (0.16)	0.25 (0.08; 0.33)	0.01 (−0.15; 0.16)	0.93
	1‐year FU	0.20 (0.29)	0.08 (0.00; 0.25)	0.22 (0.27)	0.17 (0.08; 0.17)	0.02 (−0.14; 0.18)	0.78
	3‐year FU	0.28 (0.28)	0.25 (0.00; 0.50)	0.28 (0.20)	0.28 (0.17; 0.42)	0–00 (−0.16; 0.16)	0.98
	5‐year FU	0.50 (0.29)	0.50 (0.33; 0.67)	0.56 (0.23)	0.50 (0.50; 0.75)	0.05 (−0.12; 0.22)	0.58
	7‐year FU	0.44 (0.28)	0.50 (0.25; 0.58)	0.59 (0.30)	0.63 (0.42; 0.85)	0.09 (−0.09; 0.27)	0.31
Marginal bone level	Baseline	1.28 (0.77)	1.26 (1.14; 1.92)	1.71 (0.58)	1.80 (1.42; 2.17)	0.42 (−0.09; 0.93)	0.10
	6‐month FU	1.46 (0.74)	1.38 (0.99; 1.95)	1.91 (0.68)	1.85 (2.30; 2.46)	0.44 (−0.63; 0.95)	0.09
	1‐year FU	1.33 (0.79)	1.38 (0.89; 1.83)	1.90 (0.74)	1.69 (1.41; 2.21)	0.55 (0.39; 1.06)	0.04
	3‐year FU	1.63 (0.88)	1.78 (1.12; 1.88)	2.13 (0.68)	1.95 (1.62; 2.43)	0.45 (−0.98; 0.96)	0.09
	5‐year FU	1.53 (0.73)	1.36 (1.12; 1.99)	1.79 (0.78)	1.70 (1.35; 2.14)	0.33 (−0.20; 0.87)	0.22
	7‐year FU	1.76 (0.83)	1.73 (1.18; 1.95)	1.65 (0.74)	1.62 (1.28; 2.25)	−0.16 (−0.71; 0.39)	0.57

Abbreviation: FU, follow‐up.

### 3.4 Radiographic Results

Radiographic outcomes (MBLs) are reported in Table 2 and Figure S2. At 7.5 years, mean the MBL was 1.76 ± 0.83 mm in group ONE‐C and 1.65 ± 0.74 mm in group TWO (mean difference −0.16 [95% CI: −0.7 to 0.3], *p* = 0.57). A statistically significant difference in marginal bone levels was observed only at the 1‐year follow‐up (*p* = 0.04).

### 3.5 Cost Effectiveness

**FIGURE 4:**
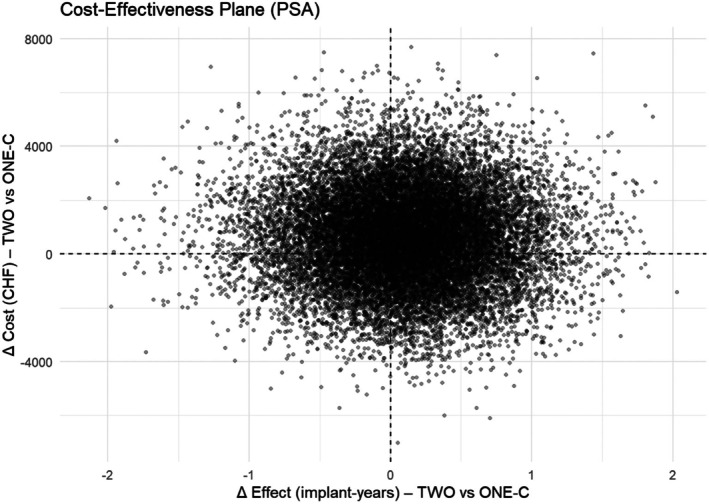
Cost‐effectiveness plane from probabilistic sensitivity analysis comparing TWO versus ONE‐C. Each dot represents one simulation. The horizontal axis shows differences in implant years and the vertical axis shows differences in cost (CHF). The wide spread of points across all four quadrants indicates no clearly dominant strategy; outcomes vary from TWO being more effective and more costly to less effective and less costly. When differences occurred, TWO generally offered slightly longer implant survival at a higher cost, with ONE‐C remaining the more cost‐effective option at common willingness‐to‐pay thresholds.

The probabilistic sensitivity analysis is presented in the cost‐effectiveness plane (Figure 4). Each dot represents one possible outcome comparing TWO versus ONE‐C in terms of cost and implant‐years. Most results cluster near the centre, suggesting that the two strategies often perform similarly. When differences did appear, TWO tended to provide slightly longer implant survival but usually at a higher cost. Across simulations, TWO was both more effective and less costly in about 21% of cases but less effective and more costly in about 27%. In 38% of simulations, TWO was more effective but also more expensive, while in 14% it was less effective but less costly. Overall, ONE‐C emerged as the more cost‐effective strategy at typical willingness‐to‐pay thresholds, since the small survival advantage of TWO often did not justify its higher cost. TWO became a cost‐effective option only when the decision‐maker was willing to pay approximately CHF 4500–5000 or more per additional implant‐year gained.

## 4 Discussion

This 7.5‐year RCT comparing one short implant with a cantilever (ONE‐C) against two short implants supporting single crowns (TWO) for the rehabilitation of posterior edentulous regions predominantly revealed the following:

1. There are no significant differences in implant survival between the two groups.
2. ONE‐C is a more cost‐effective option but with higher technical complications.
3. There is greater prevalence of peri‐implant mucositis in group TWO.
4. The clinical and radiographic outcomes are comparable over time.

### 4.1 Implant Survival

Survival rates were similar between groups (83.3% ONE‐C vs. 86.6% TWO), consistent with prior reviews showing no clear survival disadvantage for cantilever restorations (Kondo et al. 2024). However, both groups showed lower survival than typically reported for standard‐length implants (> 95% at 10 years) (Hjalmarsson et al. 2016; Howe et al. 2019; Jung et al. 2012; Papaspyridakos et al. 2018; Ravida et al. 2019). This discrepancy may be related to the use of 6‐mm‐long implants and the specific implant macro‐design used. Notably, the implants used here shared the same macro‐geometry as longer implants, with a thread pitch of 1.25 mm. For 6‐mm implants, this results in fewer threads and therefore a reduced bone‐to‐implant contact area, which may negatively affect osseointegration (Kreve et al. 2024).

Short implants have traditionally been associated with increased failure risk, although findings vary depending on anatomical location. In the atrophic maxilla, their survival is comparable or even superior to longer implants placed with sinus augmentation (Carosi et al. 2021; Fan et al. 2017; Thoma, Zeltner, et al. 2015). In contrast, results in the posterior mandible are less predictable (Papaspyridakos et al. 2018). A 5‐year RCT reported slightly lower survival for 6‐mm versus 11‐mm mandibular implants (96.0% vs. 98.9%) (Gulje et al. 2021) and another trial using the same system as the present study showed a marked difference (91.0% for 6‐mm implants vs. 100% for 10‐mm implants), with all failures in the mandible (Naenni et al. 2018).

Interestingly, implant failures were not preceded by biological complications (i.e., peri‐implantitis) or major technical complications (i.e., implant fracture) but occurred as a sudden loss of osseointegration, often reported as unexpected mobility. Similar patterns have been described with the same implant system (Naenni et al. 2018; Rossi et al. 2016) and are commonly attributed to prosthetic overload and limited bone capacity to absorb occlusal forces, especially in the posterior mandible (Gil et al. 2022; Sahrmann et al. 2017). Failures appeared earlier in the ONE‐C group, supporting the idea that two implants provide a more favourable load distribution. Radiographic data suggest that cantilevered maxillary implants may adapt to increased bone density, while mandibular sites remain more vulnerable (Gil et al. 2022). Biomechanical studies confirm that a single implant with a cantilever is exposed to higher bending forces, whereas adding a second implant reduces stress on the implant and the surrounding bone (Akça and Iplikçioğlu 2002). In this study, most ONE‐C reconstructions were free‐ending without distal tooth support, which likely increased prosthetic load and contributed to the earlier and more frequent failures observed in this group.

### 4.2 Technical Complications

Over the 7.5‐year period, 64% of all technical complications occurred in the ONE‐C group, mainly ceramic chipping (57%) and screw loosening (23%). These findings are consistent with systematic reviews reporting a higher rate of technical complications in cantilever implant–supported fixed restorations (da Freitas Silva et al. 2018; Kondo et al. 2024; Torrecillas‐Martínez et al. 2014). However, most of these studies included multi‐unit restorations, limiting their applicability to single‐implant cantilevers. A systematic review specifically addressing single‐implant cantilevers found the available evidence insufficient for reaching firm conclusions (Storelli et al. 2018), while another found that technical complications are more frequent in the posterior region (Van Nimwegen et al. 2017), consistent with the results observed in group ONE‐C.

All restorations in this study were PFM, the gold standard when the trial was initiated, which likely contributed to the high rate of ceramic chipping (Sailer et al. 2022). With advances in CAD‐CAM, monolithic all‐ceramic crowns have become reliable alternatives (Kim et al. 2023; Sailer et al. 2022), which reduce the risk of veneer fracture (Kim et al. 2023; B. E. Pjetursson et al. 2021; Sailer et al. 2022). A recent proof ‐of‐principle study demonstrated the short‐term reliability of posterior monolithic ceramic crowns with a cantilever extension, supporting their validity as a treatment option (Roccuzzo, Morandini, et al. 2023).

### 4.3 Marginal Bone Levels

While MBL was initially higher in the TWO group up to the 5‐year timepoint, the difference was no longer present at 7.5 years. The results suggest that cantilevers do not significantly affect MBL, consistent with prior clinical studies (Roccuzzo et al. 2020; Schmid et al. 2021) and systematic reviews (Aglietta et al. 2009; Kondo et al. 2024). The small differences observed may reflect measurement bias, radiographic variability or the exclusion of failed implants with low MBL, which could shift the mean values.

### 4.4 Peri‐Implant Diseases

At 7.5 years, peri‐implant mucositis was observed in 47% of patients in the ONE‐C group and 67% in the TWO group, consistent with other RCTs (Gadzo et al. 2023; Thoma et al. 2021) and systematic reviews (Derks and Tomasi 2015; Schwarz et al. 2018). The higher prevalence in TWO may be related to the larger number of implants and increased surface area for biofilm accumulation, although this remains speculative given limited evidence. The overall high prevalence may also reflect variability in diagnostic methods, as BOP is highly technique‐sensitive and influenced by probing force, angulation and instrument design (Monje and Salvi 2024). Moreover, BOP is not a definitive indicator of disease, because even small increases in probing force can induce bleeding in the absence of pathology (Gerber et al. 2009).

### 4.5 Cost Effectiveness

In our 7.5‐year analysis, using a Swiss private‐payer perspective, ONE‐C was usually more cost effective than TWO. Two implants gave only a very small extra gain in implant survival (about 0.1 years, or 1–2 months) but cost about CHF 650 more, meaning the extra cost was only worthwhile if decision makers were willing to pay over ~CHF 4500–5000 for each extra year of implant function. Other studies show similar patterns but in different settings. Zitzmann et al. found that single anterior implants were often more cost effective than bridges (Zitzmann et al. 2013). Studies of long‐term maintenance (Karlsson et al. 2022; Pirc et al. 2025) found that ongoing follow‐up and complication management can add substantial costs—up to €900–1200 over 8–10 years, or about 9% of the initial treatment cost per year. These findings matter because in our trial, TWO has higher initial cost but fewer complications, while ONE‐C has lower upfront cost but more maintenance events. This balance explains why the ‘most cost‐effective’ choice depends both on the willingness to pay and how much weight is given to complication‐related follow‐up costs.

In this study, patients in group TWO received two adjacent single crowns. Whether adjacent implants should be splinted or restored individually is being debated (Pascoal et al. 2025). In vitro studies suggest splinting distributes occlusal forces more evenly (Bergkvist et al. 2008; de Souza Batista et al. 2017), but clinical trials show no consistent differences in bone loss, survival or complications between splinted and non‐splinted crowns (Clelland et al. 2016; de Souza Batista et al. 2019; Li et al. 2022; Ravida et al. 2018). Splinting may improve contact stability, whereas single crowns facilitate hygiene and allow more predictable passive fit (de Souza Batista et al. 2019).

The main strength of this study is its cost‐effectiveness analysis, which is rarely performed in implant dentistry and therefore provides a valuable benchmark for decision making and future RCTs. This study is limited by its small sample size, with only 25 of 36 patients completing 7.5 years, reducing power for secondary outcomes. Potential effect modifiers such as implant location, cantilever orientation, parafunction and free‐ending status were not stratified; notably, all failures occurred in the mandible, suggesting site‐specific risks. Another limitation is the use of PFM crowns, which likely contributed to the high chipping rate. Modern monolithic ceramics show lower fracture risk, so our complication rates may overestimate those expected with current materials (Pjetursson et al. 2023).

## 5 Conclusion

Both treatment strategies achieved similar clinical and radiographic outcomes. Short implants with cantilever restorations were generally more cost effective than two short implants but showed higher early complication and failure rates, likely due to mechanical overload. The optimal choice therefore depends on the decision maker's willingness to pay and the importance placed on long‐term maintenance costs.

## Author Contributions

All authors made substantial contributions to this study. D.S.T. and R.J. contributed to the conception and design of the study. F.J.S., L.S., N.N., R.K. and N.M. contributed to the clinical phases of the study and collected the data. F.J.S. and G.S.R. interpreted the data and performed the statistical analysis. F.J.S., L.S. and G.S.R. drafted the manuscript and N.N., R.K., N.M., R.J., N.N. and D.S.T. critically reviewed and revised it.

## Conflicts of Interest

The authors declare no conflicts of interest.

## Supporting information


**Data S1:** Supporting Information.

## Data Availability

The data that support the findings of this study are available from the corresponding author upon reasonable request.
