# Supplementary material for: Cost Effectiveness of Two Short Implants Versus One Short Implant With a Cantilever in the Posterior Region: 7.5‐Year Follow‐Up of a Randomised Controlled Trial
Source: J Clin Periodontol. 2025 Sep 21;52(12):1712–24. doi: 10.1111/jcpe.70039 (PMC12605678; doi:10.1111/jcpe.70039)
Supplement: Supplementary file 1 — Data S1: Supporting Information. [file JCPE-52-1712-s001.zip › jcpe70039-sup-0002-Supinfo2@Supplement_cost-effectiveness analysis .pdf]

Health Economics Methods Supplement File

Cost-effectivenes of two short implants versus one short implant with a cantilever in the posterior region: 7.5-Year follow-up of a randomized controlled trial

Franz J. Strauss, Lucia Schiavon, Nadja Naenni, Riccardo D. Kraus, Gustavo Sáenz-Ravello, Nicolas Müller, Ronald E. Jung, Daniel S.Thoma

Methods

The economic evaluation followed the Consolidated Health Economic Evaluation Reporting Standards (CHEERS) 2022.[1] The interactive version is available on https://rpubs.com/gustavosaenzravello/1338253

Decision problem and comparators

We evaluated two strategies for implant-supported rehabilitation, **ONE-C** (one implant with cantilever) versus **TWO** (two implants), in adults requiring fixed rehabilitation of a posterior edentulous span.

Study perspective, setting, and audience

Primary analysis from the **Swiss private-payer/health-care system** perspective; results are intended for clinicians, payers, and guideline developers.

Time horizon and discounting

Costs and effects were accumulated over **7.5 years** and **discounted at 3% per annum** for both outcomes and costs.

Model structure

A three-state **Markov individual-level microsimulation** (base case)[2] was implemented with states: *functional*, *complication*, and *failure* (absorbing). **Annual cycles** with a **half-cycle correction** were used. Structural assumptions: (i) failure is absorbing; (ii) complications carry temporary effectiveness decrements but do not change long-run survival beyond their estimated effect on failure hazards. **Cycle:** 6 months (15 cycles in total). **States:** H (functional), C (complication; 1-cycle tunnel), F (failure; absorbing) (**Figure 1**).

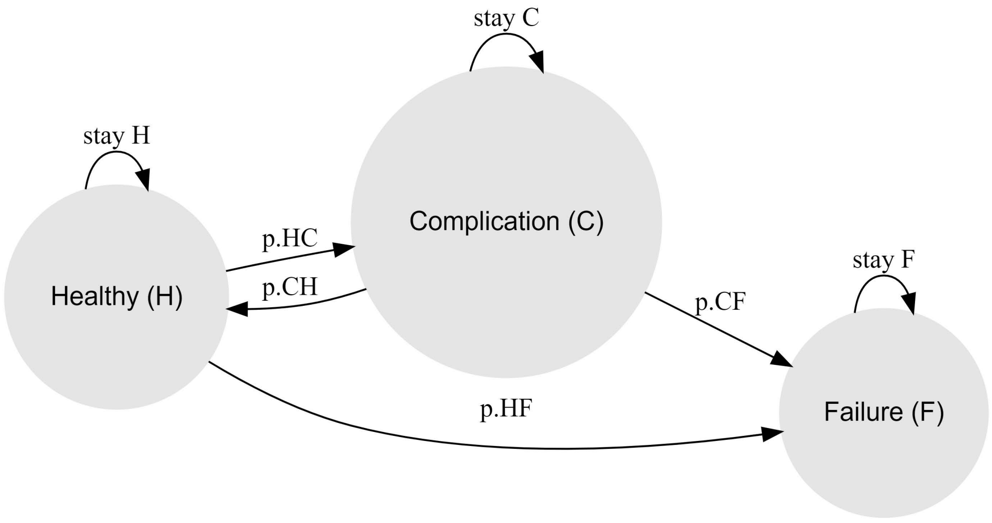

Figure 1. Schematic representation of the microsimulation model.

- **States transitions**
- **Events from H:** Complication (C) or failure (F) in the same cycle, modeled with competing risks using cause-specific hazards per year (λ_C, λ_F).
  Transition probabilities for half-year cycles:

$$p_{HC} = \frac{\lambda_C}{\lambda_C + \lambda_F}\left(1 - e^{-(\lambda_C+\lambda_F)\cdot 0.5}\right), \quad p_{HF} = \frac{\lambda_F}{\lambda_C + \lambda_F}\left(1 - e^{-(\lambda_C+\lambda_F)\cdot 0.5}\right)$$

- **Events from C (1-cycle tunnel):** Either resolution to H or failure (F) with

$$p_{CF} = 1 - e^{-\lambda_F \cdot 0.5}, \quad p_{CH} = 1 - p_{CF}$$

- **Parameterization:** Hazards (λ_C, λ_F) estimated from study data as events/person-years; mixture of complication type from relative frequencies.

Effectiveness measure

The primary outcome was **implant-years** (survival-time of the reconstruction). Temporary decrements during complication episodes were modeled as **days of impaired function**, converted to fractions of a year, and subtracted from implant-years. Accrues in H and C (0.5 per cycle). Theoretical temporary **disutility in C** parameterized by downtime in days per complication (scenario). Measured in **Implant-years** of succesful implants [3].

Identification, measurement, and valuation of outcomes and resources

Clinical inputs. Transition **hazards** (complication types and failure) and the **duration of complication-related decrements** were estimated from trial data.

Costs. We included **initial treatment** and **complication management**. Unit costs were valued in **CHF**; when price years differed, values were **inflated to the study price year using the Swiss CPI (health component)**. No currency conversion was required.

- **Initial treatment costs (implant placement and prosthetic rehabilitation):** ONE-C = 5754.6 CHF, TWO = 6465.35 CHF
- **Complication:** one event cost per entry into C. Type is sampled (screw loosening vs chipping) using the observed mixture by group; in PSA a group-specific average cost is used. Screw loosening = CHF 88.35; Chipping = CHF 55.8.
- **Failure:** no cost was applied on entry to F (from H or C).
- *No maintenance cost in H* (assumed for both groups).

Willingness-to-pay thresholds: Estimated from previous published data: CHF 2536.015, 4046.830, 4586.410, 8093.665, 12140.495

Analytic methods

We computed mean discounted **costs**, **implant-years**, and the **incremental cost-effectiveness ratio (ICER)** as ΔCost/ΔEffect (CHF per implant-year). We also calculated **net monetary benefit (NMB)** across **willingness-to-pay (WTP) per implant-year** derived from published sources (Appendix).

Uncertainty analysis

Deterministic (one-way). We varied the **duration of the complication-related disutility** (0, 7, 14, 28 days) and summarized effects on **ICER** and **NMB** across WTP values.

Probabilistic. Parameter uncertainty was propagated via **20,000 Monte Carlo simulations**. We drew **hazards and cost parameters from Gamma distributions** and **complication-type probabilities from Beta distributions**; correlations reflected shared data sources when applicable. Outputs included **CE plane**, **CEAC**, and **PSA NMB**.

Scenario analysis. Because base-case ΔEffect was small, we also performed a **cost-minimization analysis (CMA)** reporting the **incremental cost** (TWO vs ONE-C) and **Pr(TWO less costly)**.

Heterogeneity and subgroups

No a priori subgroups were powered in the trial; heterogeneity was not modeled. (If relevant subgroups become available, analyses will be pre-specified and reported.)

Model validation

Face validity was assessed with clinical experts; **internal validity** used unit-tests, extreme-value checks, and convergence diagnostics for simulation size; **cross-validation** compared model-generated survivals and event rates with observed trial summaries.

1) Packages

```
options(repos = c(CRAN = "https://cloud.r-project.org"))

if (!requireNamespace("pacman", quietly = TRUE)) {
  install.packages("pacman", dependencies = TRUE)
}

suppressPackageStartupMessages(
  pacman::p_load(readxl, dplyr, tidyr, ggplot2, classInt, Ckmeans.1d.dp, dplyr, tibble)
)
```

2) Data and Global Settings

```
# Parameters
DISCOUNT      <- params$discount
CYCLE_LEN     <- params$cycle_len
HORIZON_YEARS <- params$horizon_years
N_CYCLES      <- as.integer(HORIZON_YEARS / CYCLE_LEN)
N_MICRO       <- params$n_micro
N_PSA         <- params$n_psa
DISUTIL_DAYS  <- params$disutil_days_per_comp

# Read data
```

```r
set.seed(888)
df_raw <- read_xlsx(params$data_file, sheet = params$sheet)

infer_min_pos <- function(x, fallback = NA_real_) {
  v <- x
  v <- v[!is.na(v) & v > 0]
  if (length(v)) min(v) else fallback
}

COST_SCREW   <- infer_min_pos(df_raw$screw_loosening, fallback = 88.35)
COST_CHIP    <- infer_min_pos(df_raw$chipping,        fallback = 55.8)
COST_RETREAT <- 0

# Build df
df <- df_raw %>%
  mutate(
    events_screw = round(replace_na(screw_loosening, 0) / COST_SCREW),
    events_chip  = round(replace_na(chipping, 0) / COST_CHIP),
    py           = replace_na(`_t`, 0)
  )

by_group <- df %>%
  group_by(group) %>%
  summarise(
    n            = n(),
    failures     = sum(replace_na(failure,0) == 1),
    py           = sum(py),
    screw_events = sum(events_screw),
    chip_events  = sum(events_chip),
    init_treat_cost = mean(treatment, na.rm = TRUE),
    .groups = "drop"
  ) %>%
  mutate(
    lambda_fail = failures / py,
    lambda_comp = (screw_events + chip_events) / py,
    p_screw     = ifelse((screw_events + chip_events) > 0,
                         screw_events / (screw_events + chip_events), 0),
    p_chip      = 1 - p_screw,
    avg_comp_cost = ifelse((screw_events + chip_events) > 0,
                           (screw_events*COST_SCREW + chip_events*COST_CHIP) /
                           (screw_events + chip_events),
                           COST_CHIP)
  )

INIT_COST_G1 <- by_group$init_treat_cost[by_group$group == 1]
INIT_COST_G2 <- by_group$init_treat_cost[by_group$group == 2]

by_group
```

```
## # A tibble: 2 x 12
##   group     n failures    py screw_events chip_events init_treat_cost
##   <dbl> <int>    <int> <dbl>        <dbl>       <dbl>           <dbl>
## 1     1    18        3  120.           10          14           5755.
```

```
## 2     2    15        2  105.            1           9           6465.
## # i 5 more variables: lambda_fail <dbl>, lambda_comp <dbl>, p_screw <dbl>,
## #   p_chip <dbl>, avg_comp_cost <dbl>
```

3) Transition Probabilities and Discount

```
halfyear_probs <- function(lc, lf, dt = CYCLE_LEN) {
  s <- lc + lf
  if (s <= 0) return(c(pHC=0, pHF=0, pCF=0, pCH=1))
  p_any <- 1 - exp(-s * dt)
  pHC   <- (lc / s) * p_any          # H -> C
  pHF   <- (lf / s) * p_any          # H -> F
  pCF   <- 1 - exp(-lf * dt)         # C -> F (tunnel)
  pCH   <- 1 - pCF                   # C -> H
  c(pHC=pHC, pHF=pHF, pCF=pCF, pCH=pCH)
}

discount_factor <- function(cycle_idx, cycles_per_year = as.integer(1/CYCLE_LEN), annual_rate = DISCOUN
  t_years <- cycle_idx / cycles_per_year
  1 / ((1 + annual_rate)^t_years)
}

# Check table (rounded)
trans_table <- by_group %>%
  rowwise() %>%
  mutate(
    pHC = halfyear_probs(lambda_comp, lambda_fail)["pHC"],
    pHF = halfyear_probs(lambda_comp, lambda_fail)["pHF"],
    pCF = halfyear_probs(lambda_comp, lambda_fail)["pCF"],
    pCH = halfyear_probs(lambda_comp, lambda_fail)["pCH"]
  ) %>%
  ungroup() %>%
  select(group, lambda_comp_per_year = lambda_comp, lambda_fail_per_year = lambda_fail,
         `P(H->C) per 6mo` = pHC, `P(H->F) per 6mo` = pHF,
         `P(C->F) per 6mo` = pCF, `P(C->H) per 6mo` = pCH)

round(trans_table, 4)
```

```
## # A tibble: 2 x 7
##   group lambda_comp_per_year lambda_fail_per_year `P(H->C) per 6mo`
##   <dbl>                <dbl>                <dbl>             <dbl>
## 1     1               0.200                0.0251            0.0948
## 2     2               0.0952               0.019             0.0463
## # i 3 more variables: `P(H->F) per 6mo` <dbl>, `P(C->F) per 6mo` <dbl>,
## #   `P(C->H) per 6mo` <dbl>
```

4) Microsimulation Function *(with theoretical disutility in C)*

```
# states: 0=H, 1=C (1-cycle tunnel), 2=F
# If DISUTIL_DAYS > 0: when an individual enters C in a cycle, we subtract
# disutility_implant_years = min(CYCLE_LEN, DISUTIL_DAYS/365) from that individual's
# effectiveness accrual for that cycle (not below zero).
# states: 0=H, 1=C (1-cycle tunnel), 2=F
microsim_group <- function(N, lc, lf, p_mix_screw, cost_retreat,
                           cycles = N_CYCLES, cycle_len = CYCLE_LEN, discount = DISCOUNT,
                           cost_screw = COST_SCREW, cost_chip = COST_CHIP,
                           disutil_days = DISUTIL_DAYS,
                           init_cost = 0) {

  state <- rep(0L, N)
  costs <- numeric(N)
  eff   <- numeric(N)
  set.seed(888)
  disutil_implant_years <- min(cycle_len, disutil_days/365)

  # Upfront initial treatment cost at baseline (undiscounted)
  if (init_cost != 0) costs <- costs + init_cost

  for (c in 0:(cycles-1)) {
    dfac   <- discount_factor(c, cycles_per_year = as.integer(1/cycle_len), annual_rate = discount)
    probs  <- halfyear_probs(lc, lf, cycle_len)
    pHC    <- probs["pHC"]; pHF <- probs["pHF"]; pCF <- probs["pCF"]; pCH <- probs["pCH"]

    # Effect accrual for non-failed
    alive <- state != 2L
    eff[alive] <- eff[alive] + cycle_len * dfac

    # From H
    H_idx <- which(state == 0L)
    if (length(H_idx)) {
      stayH <- 1 - (pHC + pHF)
      draws <- sample(c(0,1,2), size=length(H_idx), replace=TRUE, prob=c(stayH, pHC, pHF))
      toC   <- H_idx[draws == 1]
      toF   <- H_idx[draws == 2]
      if (length(toC)) {
        comp_is_screw <- rbinom(length(toC), 1, p_mix_screw) == 1
        costs[toC] <- costs[toC] + ifelse(comp_is_screw, cost_screw, cost_chip) * dfac
        if (disutil_implant_years > 0) {
          eff[toC] <- pmax(0, eff[toC] - disutil_implant_years * dfac)
        }
        state[toC] <- 1L
      }
      if (length(toF)) {
        costs[toF] <- costs[toF] + cost_retreat * dfac  # 0 by design
        state[toF] <- 2L
      }
    }

    # C tunnel
```

```
    C_idx <- which(state == 1L)
    if (length(C_idx)) {
      u   <- runif(length(C_idx))
      toF <- C_idx[u < pCF]
      toH <- C_idx[u >= pCF]
      if (length(toF)) {
        costs[toF] <- costs[toF] + cost_retreat * dfac  # 0 by design
        state[toF] <- 2L
      }
      if (length(toH)) {
        state[toH] <- 0L
      }
    }
  }
  c(mean_cost = mean(costs), mean_implant_years = mean(eff))
}
```

5) Base-Case Simulation and ICER

```
# Extract per-group parameters
par_g1 <- by_group %>% filter(group==1)
par_g2 <- by_group %>% filter(group==2)

out_g1 <- microsim_group(
  N = N_MICRO,
  lc = par_g1$lambda_comp, lf = par_g1$lambda_fail,
  p_mix_screw = par_g1$p_screw,
  cost_retreat = COST_RETREAT,
  init_cost = INIT_COST_G1
)

out_g2 <- microsim_group(
  N = N_MICRO,
  lc = par_g2$lambda_comp, lf = par_g2$lambda_fail,
  p_mix_screw = par_g2$p_screw,
  cost_retreat = COST_RETREAT,
  init_cost = INIT_COST_G2
)

base_results <- data.frame(
  group = c(1,2),
  mean_cost = c(out_g1["mean_cost"], out_g2["mean_cost"]),
  mean_implant_years = c(out_g1["mean_implant_years"], out_g2["mean_implant_years"])
) %>% arrange(group)

g1_cost  <- base_results$mean_cost[base_results$group==1]
g1_eff   <- base_results$mean_implant_years[base_results$group==1]

base_results <- base_results %>%
  mutate(
    delta_cost_vs_g1 = mean_cost - g1_cost,
```

```
    delta_eff_vs_g1   = mean_implant_years - g1_eff,
    ICER_vs_g1        = delta_cost_vs_g1 / delta_eff_vs_g1
  )

round(base_results, 3)
```

```
##   group mean_cost mean_implant_years delta_cost_vs_g1 delta_eff_vs_g1
## 1     1  5836.352              6.216            0.000           0.000
## 2     2  6499.988              6.351          663.636           0.135
##   ICER_vs_g1
## 1        NaN
## 2   4907.486
```

In the base case, the TWO (two-implant) strategy was modestly more effective than ONE-C (single implant with cantilever), with a mean gain of 0.135 implant-years, consistent with clinical near-equivalence. Mean discounted costs were higher for TWO (CHF 6,500) than for ONE-C (CHF 5,836), yielding an incremental cost of CHF 663 (TWO vs ONE-C). The resulting ICER was CHF 4,907.5 per implant-year. Whether TWO is preferred depends on the decision-maker's willingness-to-pay per implant-year

5) Probabilistic Sensitivity Analysis (cohort level)

```
cohort_outcomes <- function(lc, lf, avg_comp_cost, cost_retreat,
                            cycles = N_CYCLES, cycle_len = CYCLE_LEN, discount = DISCOUNT,
                            init_cost = 0) {
  H <- 1.0; C <- 0.0; F <- 0.0
  total_cost <- 0.0; total_eff <- 0.0


  if (init_cost != 0) total_cost <- total_cost + init_cost

  for (c in 0:(cycles-1)) {
    dfac <- discount_factor(c, cycles_per_year = as.integer(1/cycle_len), annual_rate = discount)
    pr   <- halfyear_probs(lc, lf, cycle_len)
    pHC  <- pr["pHC"]; pHF <- pr["pHF"]; pCF <- pr["pCF"]; pCH <- pr["pCH"]

    H_to_C <- H * pHC; H_to_F <- H * pHF; H_stay <- H * (1 - pHC - pHF)
    C_to_F <- C * pCF; C_to_H <- C * (1 - pCF)

    H <- H_stay + C_to_H
    C <- H_to_C
    F <- F + H_to_F + C_to_F

    total_cost <- total_cost + (H_to_C * avg_comp_cost + (H_to_F + C_to_F) * cost_retreat) * dfac
    total_eff  <- total_eff + (H + C) * cycle_len * dfac
  }
  c(total_cost = total_cost, total_eff = total_eff)
}
```

```r
set.seed(888)

# Groups
g1 <- by_group %>% dplyr::filter(group == 1)
g2 <- by_group %>% dplyr::filter(group == 2)

# 1) Hazards ~ Gamma (Poisson-Gamma conjugacy)
lam_f_1 <- rgamma(N_PSA, shape = g1$failures + 1, rate = g1$py)
lam_c_1 <- rgamma(N_PSA, shape = (g1$screw_events + g1$chip_events) + 1, rate = g1$py)
lam_f_2 <- rgamma(N_PSA, shape = g2$failures + 1, rate = g2$py)
lam_c_2 <- rgamma(N_PSA, shape = (g2$screw_events + g2$chip_events) + 1, rate = g2$py)

# 2) Complication mix ~ Beta (p_screw); mean complication cost per iteration
a1 <- g1$screw_events + 1; b1 <- g1$chip_events + 1
a2 <- g2$screw_events + 1; b2 <- g2$chip_events + 1
p_screw_1 <- rbeta(N_PSA, a1, b1)
p_screw_2 <- rbeta(N_PSA, a2, b2)
mu_c1 <- p_screw_1 * COST_SCREW + (1 - p_screw_1) * COST_CHIP
mu_c2 <- p_screw_2 * COST_SCREW + (1 - p_screw_2) * COST_CHIP

# 3) Costs ~ Gamma with assumed CV (no empirical variance available)
cv_comp <- 0.20
sd_c1     <- pmax(1e-8, cv_comp * mu_c1)
sd_c2     <- pmax(1e-8, cv_comp * mu_c2)
shape_c1  <- (mu_c1^2) / (sd_c1^2);  rate_c1 <- mu_c1 / (sd_c1^2)
shape_c2  <- (mu_c2^2) / (sd_c2^2);  rate_c2 <- mu_c2 / (sd_c2^2)
avg_c1_draw <- rgamma(N_PSA, shape = shape_c1, rate = rate_c1)
avg_c2_draw <- rgamma(N_PSA, shape = shape_c2, rate = rate_c2)

# 4) Retreatment cost = 0 in all iterations
ret_draw <- rep(0, N_PSA)

# 5) Initial treatment cost ~ Gamma by group (include upfront)
cv_init <- 0.20
mu_init1 <- INIT_COST_G1; mu_init2 <- INIT_COST_G2
sd_init1 <- pmax(1e-8, cv_init * mu_init1); sd_init2 <- pmax(1e-8, cv_init * mu_init2)
shape_init1 <- (mu_init1^2) / (sd_init1^2); rate_init1 <- mu_init1 / (sd_init1^2)
shape_init2 <- (mu_init2^2) / (sd_init2^2); rate_init2 <- mu_init2 / (sd_init2^2)
init1_draw <- rgamma(N_PSA, shape = shape_init1, rate = rate_init1)
init2_draw <- rgamma(N_PSA, shape = shape_init2, rate = rate_init2)

# 6) Run cohort per iteration
res1 <- vapply(1:N_PSA, function(i) {
  cohort_outcomes(lc = lam_c_1[i], lf = lam_f_1[i],
                  avg_comp_cost = avg_c1_draw[i], cost_retreat = ret_draw[i],
                  cycles = N_CYCLES, cycle_len = CYCLE_LEN, discount = DISCOUNT,
                  init_cost = init1_draw[i])
}, FUN.VALUE = c(total_cost = 0, total_eff = 0))

res2 <- vapply(1:N_PSA, function(i) {
  cohort_outcomes(lc = lam_c_2[i], lf = lam_f_2[i],
                  avg_comp_cost = avg_c2_draw[i], cost_retreat = ret_draw[i],
                  cycles = N_CYCLES, cycle_len = CYCLE_LEN, discount = DISCOUNT,
```

```r
                  init_cost = init2_draw[i])
}, FUN.VALUE = c(total_cost = 0, total_eff = 0))

# 7) Incrementals and summary
psa_df <- data.frame(
  dCost_g2_vs_g1 = res2["total_cost", ] - res1["total_cost", ],
  dEff_g2_vs_g1  = res2["total_eff", ]  - res1["total_eff", ]
)
psa_df$ICER <- psa_df$dCost_g2_vs_g1 / psa_df$dEff_g2_vs_g1

summary_df <- data.frame(
  dCost_mean  = mean(psa_df$dCost_g2_vs_g1),
  dCost_sd    = sd(psa_df$dCost_g2_vs_g1),
  dEff_mean   = mean(psa_df$dEff_g2_vs_g1),
  dEff_sd     = sd(psa_df$dEff_g2_vs_g1),
  ICER_median = stats::median(psa_df$ICER, na.rm = TRUE)
)
summary_df
```

```
##   dCost_mean dCost_sd  dEff_mean  dEff_sd ICER_median
## 1   656.2102 1717.599 0.09771274 0.494284    253.6504
```

```r
# Probability TWO cheaper
Pr_Two_cheaper <- mean(psa_df$dCost_g2_vs_g1 < 0, na.rm = TRUE)
Pr_Two_cheaper
```

```
## [1] 0.3489
```

The PSA yielded a mean incremental cost of +CHF 656.2 (SD 1,717.6) and a mean incremental effect of +0.098 implant-years (SD 0.494), indicating that TWO is, on average, more effective and more costly than ONE-C, with substantial uncertainty. TWO was cheaper in 34.9% of simulations. The median ICER across simulations was ≈ CHF 254 per implant-year. Overall, these results are consistent with the base-case pattern (NE quadrant). Preference for TWO therefore depends on the decision-maker's willingness-to-pay, values around or above ~CHF 4,800 per implant-year would generally favor TWO, whereas lower values favor ONE-C.

A) Absolute Results by Group (PSA)

We display **absolute** distributions of **cost** and **effectiveness (implant-years)** for each group using the PSA samples.

```
## # A tibble: 2 x 5
##   group cost_mean cost_sd eff_mean eff_sd
##   <fct>     <dbl>   <dbl>    <dbl>  <dbl>
## 1 ONE-C     5839.   1149.     6.02  0.345
## 2 TWO       6495.   1282.     6.11  0.352
```

Mean costs were slightly lower in Group TWO (CHF 6495.1) compared with Group ONE-C (CHF 5838.89), with overlapping distributions indicating substantial variability in both groups. In terms of effectiveness, measured in implant-years, Group 2 showed a marginally higher mean (6.11 vs. 6.02) but again with overlapping distributions.

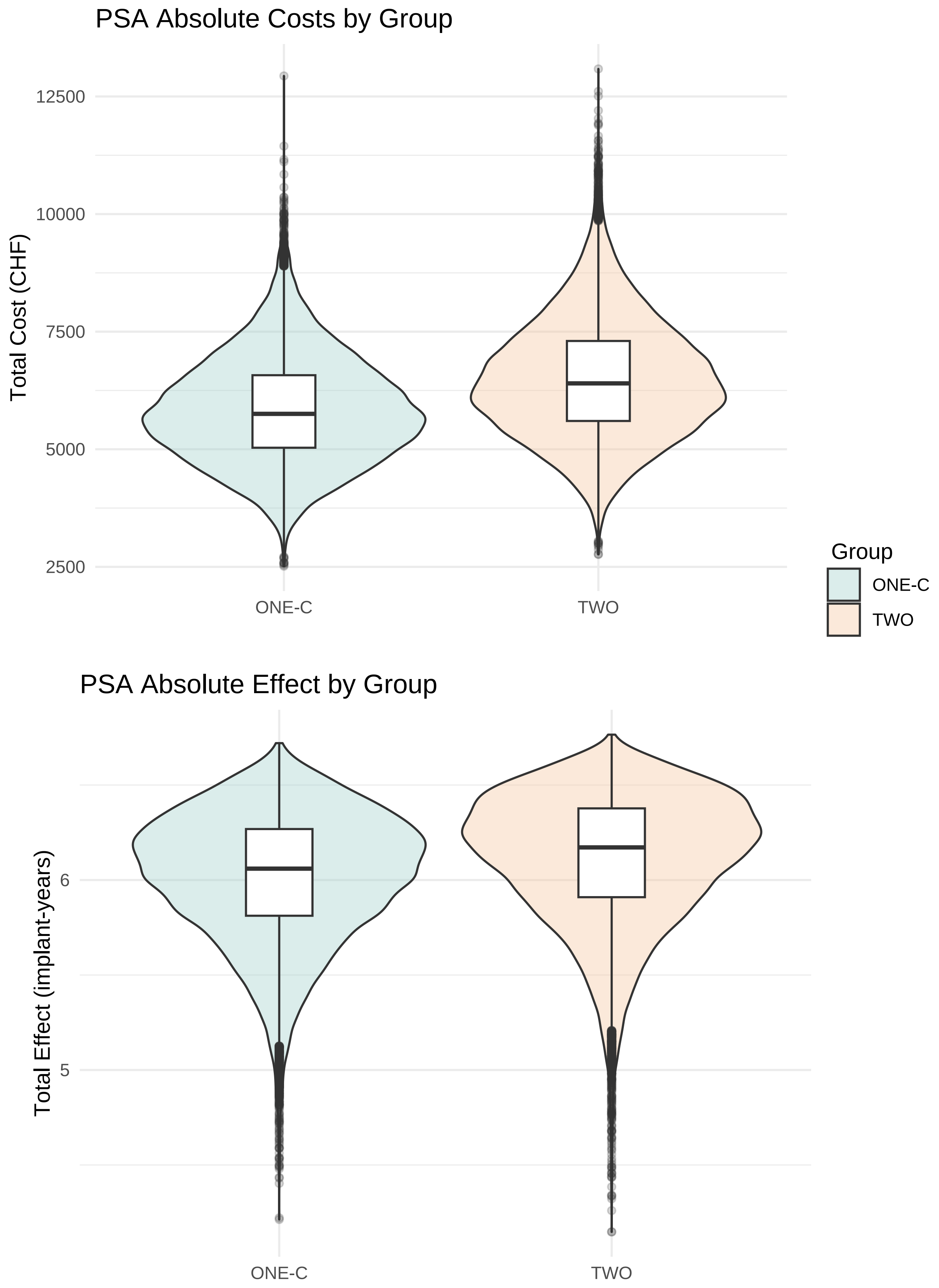

The violin plots highlight these similarities, showing that while central tendencies differ slightly, the spread and overlap suggest no large, consistent divergence in outcomes between the two strategies.

6) CE Plane and CEAC

```
##            Cost_Pos
## Effect_Pos FALSE TRUE
##      FALSE  14.0 26.9
##      TRUE   20.9 38.2
```

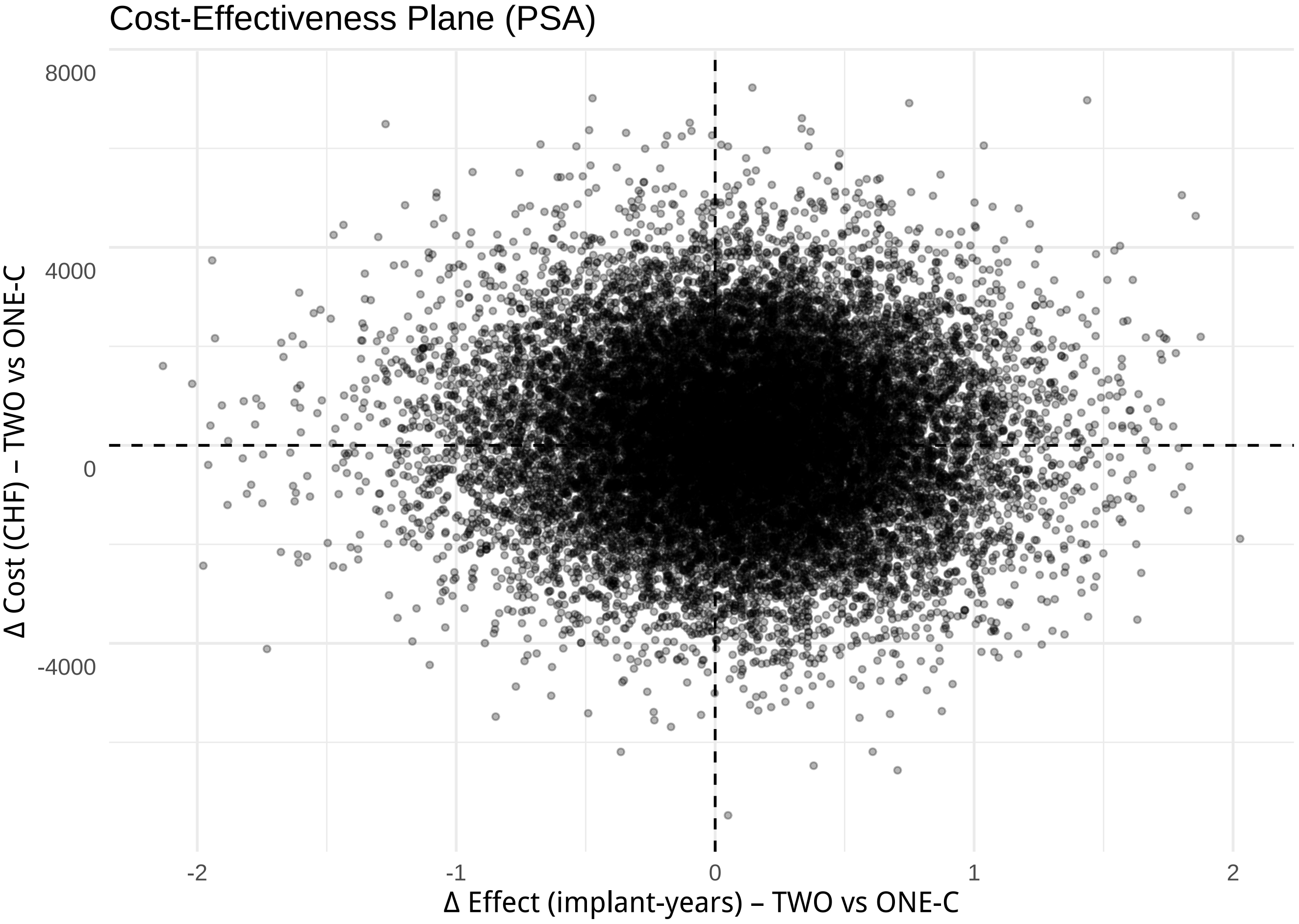

Most iterations cluster near the origin, indicating near-equivalence in many draws. There is no strong linear relationship between incremental costs and effects. If anything, gains in effectiveness often come with higher costs (largest share in the NE quadrant). Quadrant shares were: SE (ΔCost<0, ΔEff>0): 20.9%—TWO dominant; NE (ΔCost>0, ΔEff>0): 38.2%—TWO more effective and more costly; NW (ΔCost>0, ΔEff<0): 26.9%—TWO dominated; and SW (ΔCost<0, ΔEff<0): 14.0%—trade-off where preference depends on the WTP threshold. Overall, TWO is cost-saving and more effective in ~21% of simulations, dominated in ~27%, and more effective but more costly in ~38%; thus the choice between strategies hinges on the decision-maker's willingness-to-pay per implant-year.

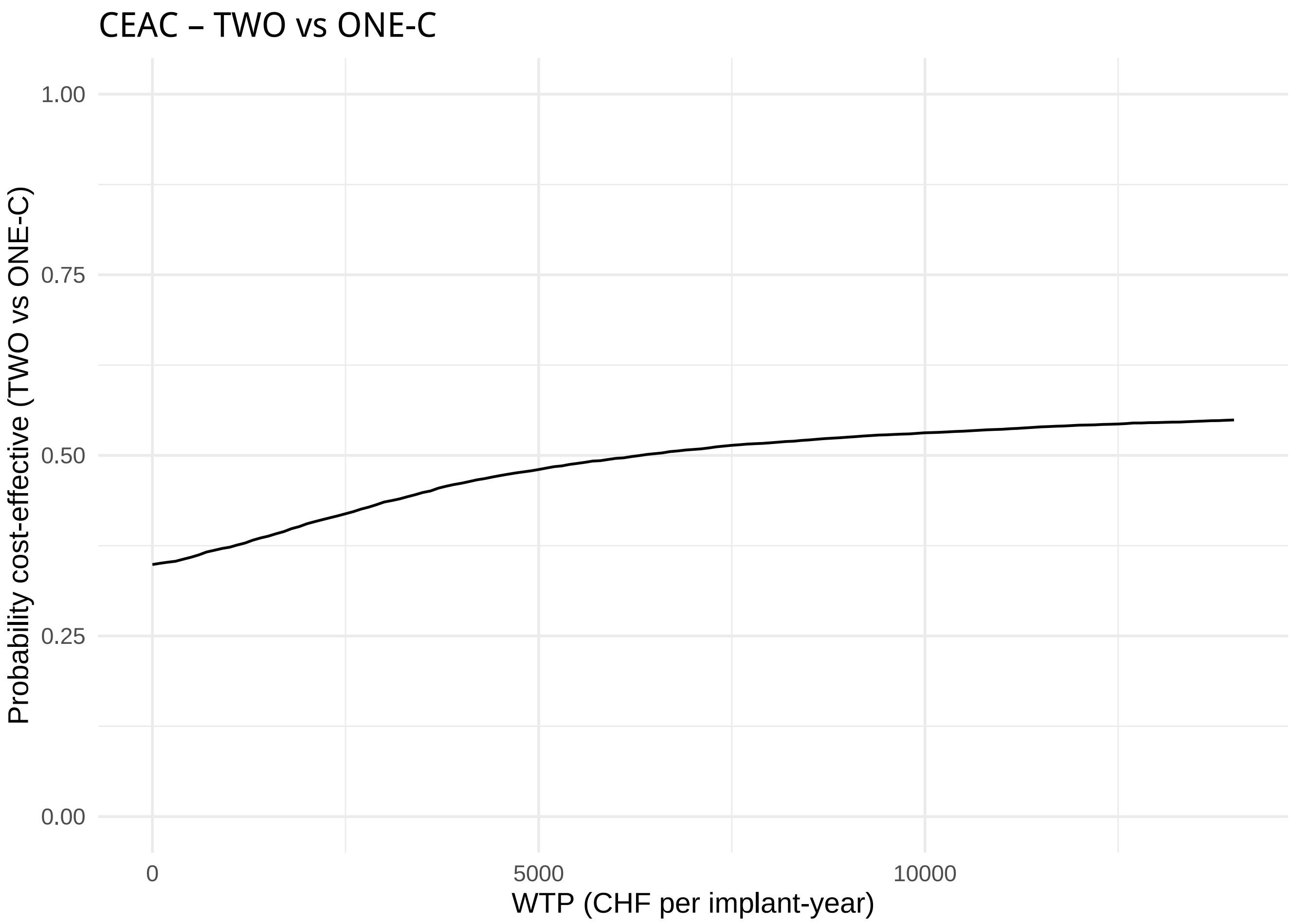

The probability that TWO is cost-effective versus ONE-C starts at ~0.35 when WTP=0 (consistent with 34.9% of PSA draws where TWO is cheaper), increases monotonically with willingness-to-pay, crosses ~0.50 around CHF 4,500–5,000 per implant-year, and reaches only ~0.53–0.55 at λ=10,000. Thus, within the evaluated range the probability never exceeds 0.60, indicating persistent decision uncertainty and near-equivalence; TWO is more likely to be preferred only at higher WTP, reflecting that it is on average more effective but also more costly.

```
## # A tibble: 5 x 6
##      WTP mean_NMB sd_NMB    q025   q975 Pr_NMB_pos
##    <dbl>    <dbl>  <dbl>   <dbl>  <dbl>      <dbl>
## 1  2536.    -408.  2152.  -4678.  3822.      0.420
## 2  4047.    -261.  2670.  -5598.  4979.      0.462
## 3  4586.    -208.  2879.  -5968.  5463.      0.474
## 4  8094.     135.  4394.  -8682.  8752.      0.518
## 5 12140.     530.  6284. -12183. 12816.      0.542
```

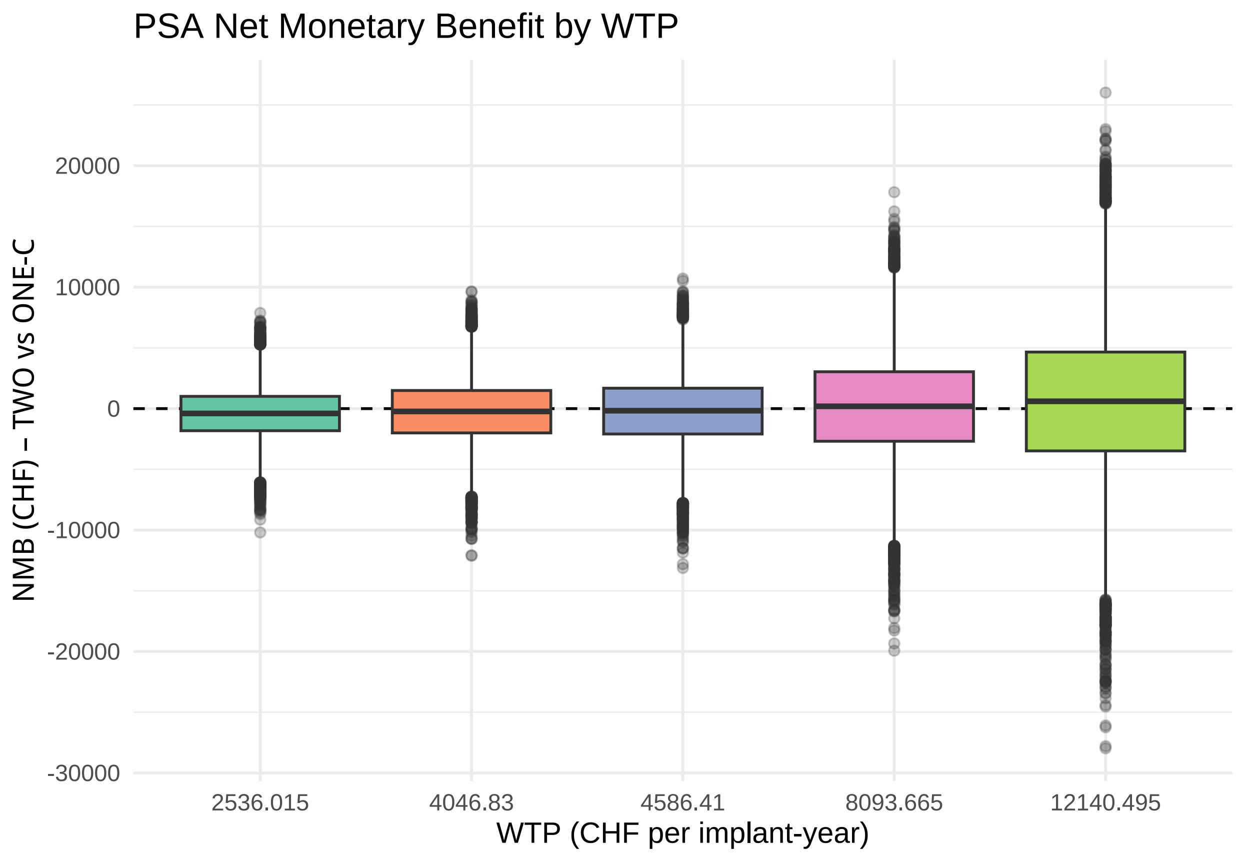

7) Disutility in C – Scenario Notes

We wanted to test a **temporary effectiveness decrement** for each complication episode, setting `params$disutil_days_per_comp` to a non-zero value, considering a theoretical yet possible reduction in the total effectiveness. The model will subtract `min(cycle_len, disutil_days/365)` implant-years in any cycle where the patient enters **C**, in addition to the cost of that complication and failure risk.

Sensitivity Analysis on Disutility in C

We vary `disutil_days_per_comp` over a plausible range (0, 7, 14, 28 days) and re-run the base-case microsimulation for each value, comparing ICERs.

```
## A) Sensitivity Analysis on Disutility in C

sens_days <- c(0, 7, 14, 28)

sens_results <- lapply(sens_days, function(ddays) {
  tmp_res <- by_group %>%
    dplyr::rowwise() %>%
    do({
      pr <- .
      out <- microsim_group(
```

```r
        N = N_MICRO,
        lc = pr$lambda_comp, lf = pr$lambda_fail,
        p_mix_screw = pr$p_screw,
        cost_retreat = COST_RETREAT,
        disutil_days = ddays,
        init_cost = pr$init_treat_cost
      )
      data.frame(
        group = pr$group,
        mean_cost = out["mean_cost"],
        mean_implant_years = out["mean_implant_years"]
      )
    }) %>%
    dplyr::ungroup() %>%
    dplyr::arrange(group)

  g1_cost <- tmp_res$mean_cost[tmp_res$group == 1]
  g1_eff  <- tmp_res$mean_implant_years[tmp_res$group == 1]

  tmp_res <- tmp_res %>%
    dplyr::mutate(
      delta_cost_vs_g1 = mean_cost - g1_cost,
      delta_eff_vs_g1  = mean_implant_years - g1_eff,
      ICER_vs_g1       = delta_cost_vs_g1 / delta_eff_vs_g1,
      disutil_days     = ddays
    )

  tmp_res[tmp_res$group == 2, c("disutil_days","delta_cost_vs_g1","delta_eff_vs_g1","ICER_vs_g1")]
}) %>% dplyr::bind_rows()

sens_results
```

```
## # A tibble: 4 x 4
##   disutil_days delta_cost_vs_g1 delta_eff_vs_g1 ICER_vs_g1
##          <dbl>            <dbl>           <dbl>      <dbl>
## 1            0             664.           0.135      4907.
## 2            7             664.           0.147      4527.
## 3           14             664.           0.158      4202.
## 4           28             664.           0.181      3674.
```

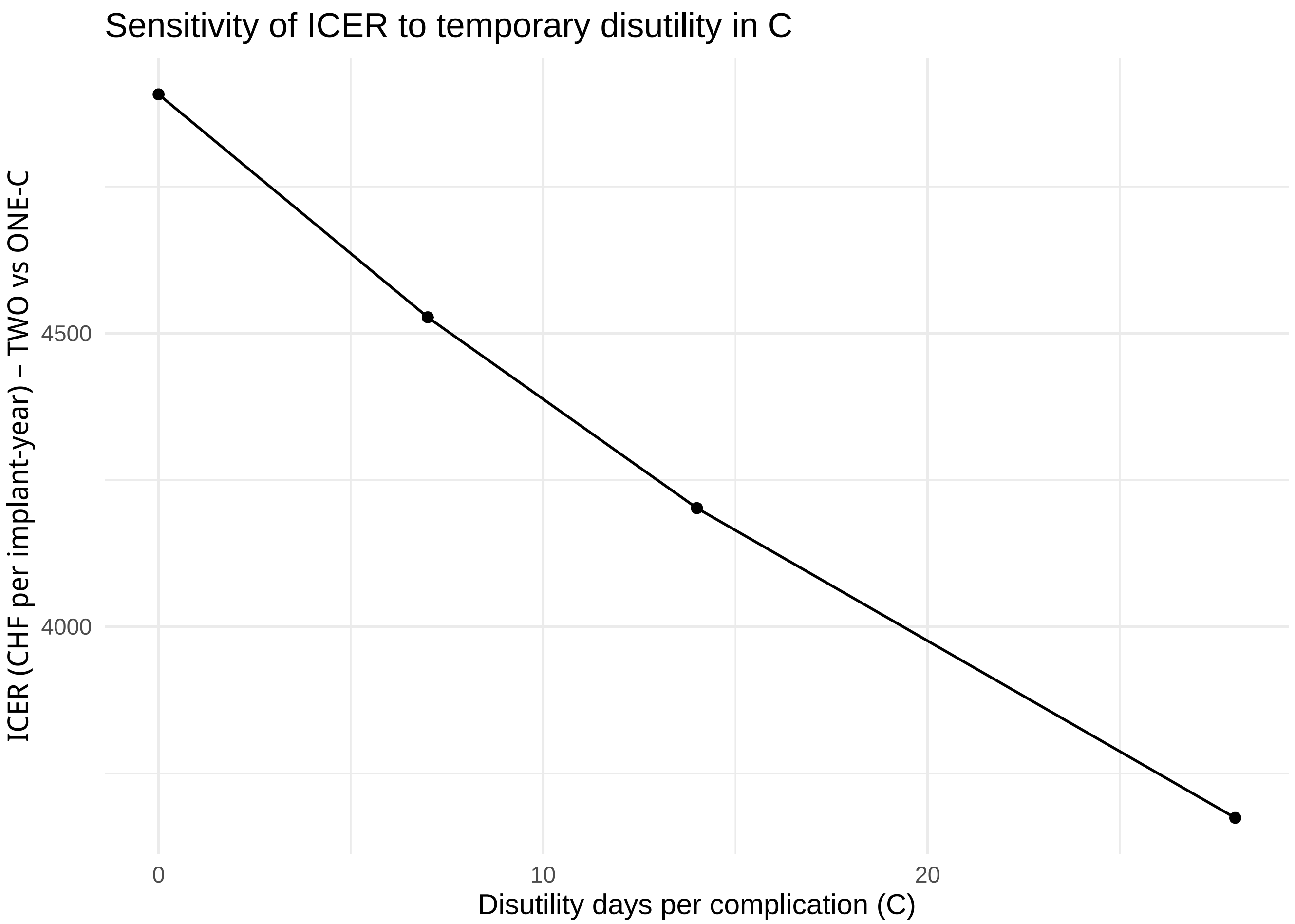

Varying the temporary disutility from 0 → 7 → 14 → 28 days makes TWO progressively more cost-effective while remaining in the NE quadrant (more effective, more costly) throughout. With ΔCost held constant at +CHF 663.72, ΔEffect rises from 0.1393 to 0.1506, 0.1620, and 0.1846 implant-years, and the ICER falls from CHF 4,765.6 to 4,406.8, 4,098.2, and 3,594.8 per implant-year. Overall, the ICER drops by about CHF 1,171 across 0–28 days—approximately -42 CHF per additional disutility day—because higher short-term losses after complications penalize ONE-C's effectiveness relatively more while costs change little. At a WTP of ~CHF 4,000 per implant-year, TWO becomes cost-effective when disutility is ≈17 days per complication or more; at higher WTP thresholds (e.g., CHF 5,000), TWO is cost-effective across all tested values.

Net Monetary Benefit vs Disutility (Selected WTPs)

We compute **deterministic** Net Monetary Benefit difference (TWO vs ONE-C) as `NMB = WTP*ΔE - ΔC` for a few WTP values.

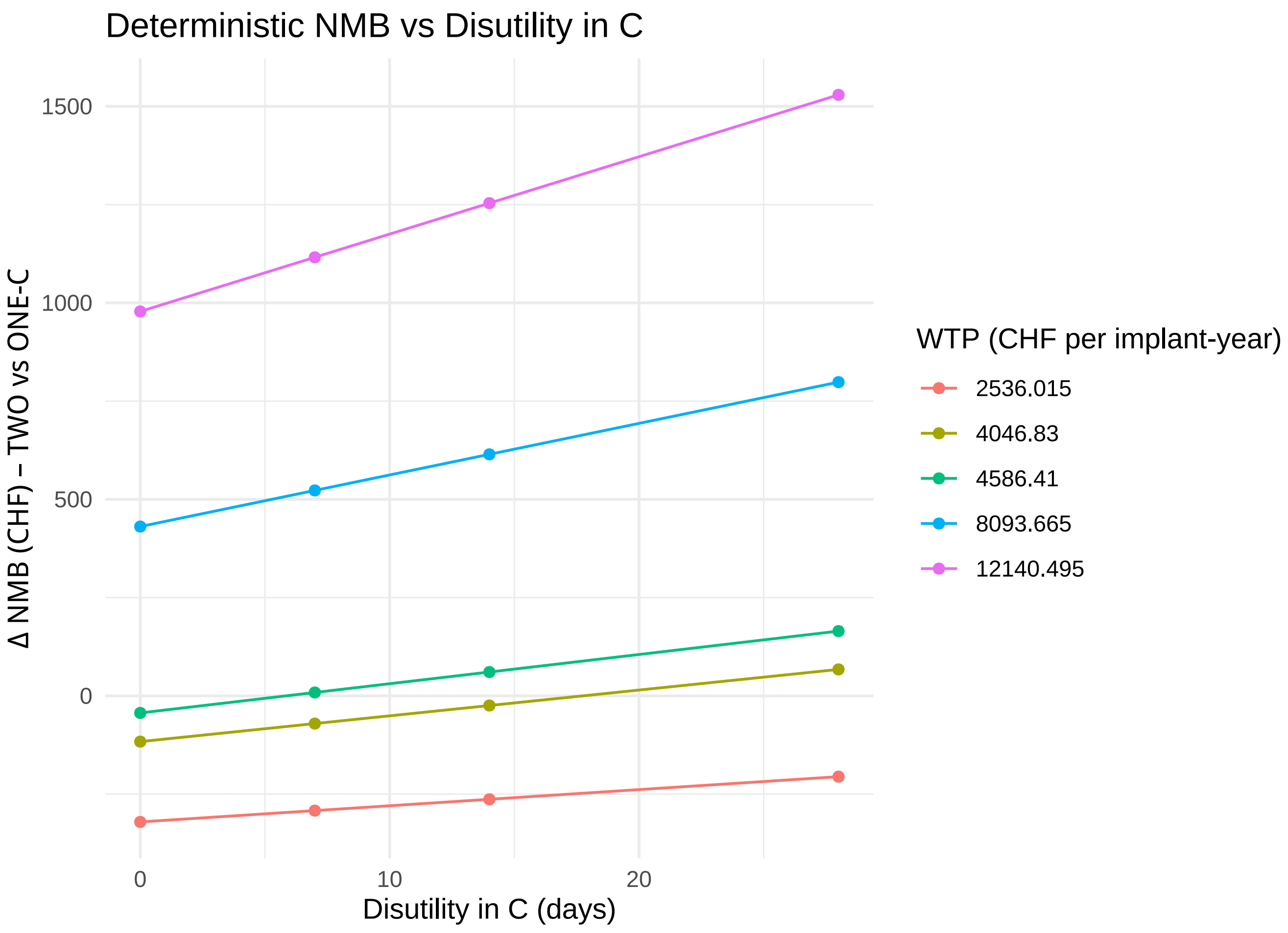

As the assumed disutility per complication increases from 0 → 7 → 14 → 28 days, ΔEffect rises (0.139 → 0.151 → 0.162 → 0.185 implant-years), so ΔNMB increases near-linearly for any WTP (λ). Using WTP in 2025 CHF, at $\lambda = 2{,}536$ ΔNMB remains negative over all disutilities (≈ -310 → -196 CHF). At $\lambda = 4{,}047$, ΔNMB becomes positive between 14 and 28 days (≈ -100 → +84 CHF). At $\lambda = 4{,}586$, it is already positive from 7 days onward (≈ -25 → +183 CHF). For higher WTPs—$\lambda = 8{,}094$ and 12,140—TWO is cost-effective for all disutilities, with ΔNMB increasing from ≈ 464 → 831 CHF and 1,027 → 1,578 CHF, respectively, as disutility rises to 28 days. Consistent with the break-even ICER thresholds in 2025 CHF (≈ 5,150, 4,740, 4,420, and 3,880 CHF per implant-year for 0, 7, 14, and 28 days), TWO is preferred only when WTP exceeds the relevant threshold, with a stronger advantage as greater short-term functional loss after complications is assumed.

8) Cost-minimization analysis

Furthermore, **assuming equivalent effectiveness**, we conducted a CMA. In the base case, mean per-patient costs were 5,836.35 CHF for ONE-C and 6,499.99 CHF for TWO, yielding an incremental cost (TWO vs ONE-C) of +663.64 CHF on average. The probabilistic sensitivity analysis (PSA) propagated parameter uncertainty and produced a distribution of incremental costs (ΔCosts) centered slightly above zero (mean +656.21 CHF; SD 1,717.60 CHF). Thirty-five percent of simulations fell below zero, indicating a non-negligible probability that TWO is

cost-saving relative to ONE-C. The 95% uncertainty interval ranged from -2,702.92 to +4,100.35 CHF, underscoring substantial uncertainty around the cost difference.

```
## [1] "Results from base case"

##   cost_onec cost_two delta_cost_two_vs_onec
## 1   5836.35  6499.99                 663.64

## [1] "Results from PSA"

##      dC_mean  dC_sd  dC_q025 dC_q975 Pr_Two_cheaper
## 2.5%  656.21 1717.6 -2702.92 4100.35           0.35
```

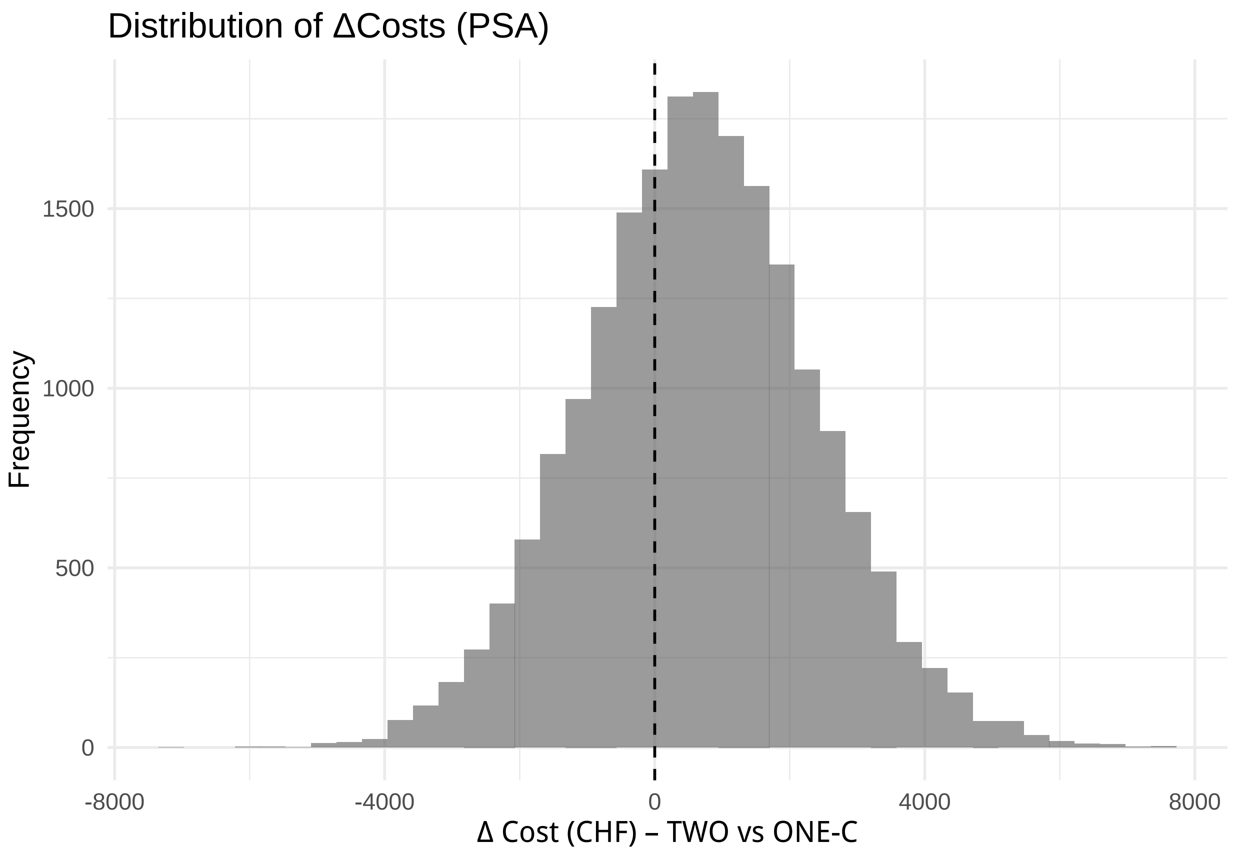

Appendix: Willingness-to-pay thresholds

```
## ===== Setup =====
library(classInt)
library(Ckmeans.1d.dp)
library(dplyr)
library(tibble)
```

```r
## ===== Data =====
x  <- c(1500,1700,3000,3000,3400,3500,4000,4000,4000,4000,4500,5000,5000,10000,12500)
xs <- sort(x)
K  <- 6   # 6 groups => 5 cuts

## ===== Currency adjustment =====

idx_2017 <- 100.0968   # OECD/FRED annual 2017
idx_2025_jul <- 108.02 # Eurostat/FRED Jul-2025

inflator <- idx_2025_jul / idx_2017
inflator
```

```
## [1] 1.079155
```

```r
# WTP ajustados a CHF 2025 (jul-2025)
x_2025 <- round(x * inflator, 2)
xs <- sort(x_2025)

## ===== Helper =====
label_with_cuts <- function(x, cuts, digits = 0) {
  brks <- c(-Inf, sort(unique(cuts)), Inf)
  fmt  <- function(v) ifelse(is.infinite(v),
                             ifelse(v < 0, "-Inf", "Inf"),
                             format(round(v, digits), nsmall = digits,
                                    scientific = FALSE, trim = TRUE))
  lefts  <- head(brks, -1)
  rights <- tail(brks, -1)
  labs <- paste0("(", fmt(lefts), ", ", fmt(rights), "]")
  labs[1] <- paste0("[", fmt(lefts[1]), ", ", fmt(rights[1]), "]")
  cut(x, breaks = brks, include.lowest = TRUE, right = TRUE, labels = labs)
}

## ===== 1) Fisher-Jenks =====
fj <- classIntervals(xs, n = K, style = "fisher")
cuts_fisher <- fj$brks[-c(1, length(fj$brks))]

## ===== 2) Ckmeans 1D =====
ckmeans_cuts <- function(xs, K, min_size = NULL) {

  if (!is.null(min_size) && "min.size" %in% names(formals(Ckmeans.1d.dp))) {
    ck <- Ckmeans.1d.dp(xs, k = K, min.size = min_size)
  } else {
    ck <- Ckmeans.1d.dp(xs, k = K)
  }
  idx  <- cumsum(ck$size)[1:(K-1)]
  (xs[idx] + xs[idx + 1]) / 2
}
cuts_ck <- ckmeans_cuts(xs, K)

## ===== 3) Largest Gaps =====
largest_gaps_cuts <- function(xs, K) {
  d <- diff(xs)
```

```
  idx_top <- order(d, decreasing = TRUE)[1:(K-1)]
  sort( (xs[idx_top] + xs[idx_top + 1]) / 2 )
}
cuts_gaps <- largest_gaps_cuts(xs, K)

## ===== Cuts for WTP thresholds =====
cuts_fisher
```

```
## [1]  2536.015  4046.830  4586.410  8093.665 12140.495
```

```
cuts_ck
```

```
## [1]  2536.015  4046.830  4586.410  8093.665 12140.495
```

```
cuts_gaps
```

```
## [1]  2536.015  4046.830  4586.410  8093.665 12140.495
```

```
## ===== Labels =====
gr_fisher <- label_with_cuts(x, cuts_fisher)
gr_ck     <- label_with_cuts(x, cuts_ck)
gr_gaps   <- label_with_cuts(x, cuts_gaps)

cbind(WTP = x,
      Fisher_Jenks = gr_fisher,
      Ckmeans_1D   = gr_ck,
      Largest_Gaps = gr_gaps)
```

```
##         WTP Fisher_Jenks Ckmeans_1D Largest_Gaps
##  [1,]  1500            1          1            1
##  [2,]  1700            1          1            1
##  [3,]  3000            2          2            2
##  [4,]  3000            2          2            2
##  [5,]  3400            2          2            2
##  [6,]  3500            2          2            2
##  [7,]  4000            2          2            2
##  [8,]  4000            2          2            2
##  [9,]  4000            2          2            2
## [10,]  4000            2          2            2
## [11,]  4500            3          3            3
## [12,]  5000            4          4            4
## [13,]  5000            4          4            4
## [14,] 10000            5          5            5
## [15,] 12500            6          6            6
```

```
## ===== groups sizes =====
table(gr_fisher); table(gr_ck); table(gr_gaps)
```

```
## gr_fisher
##  [-Inf, 2536]  (2536, 4047]  (4047, 4586]  (4586, 8094] (8094, 12140]
##             2             8             1             2             1
##  (12140, Inf]
##             1
```

```
## gr_ck
##  [-Inf, 2536]  (2536, 4047]  (4047, 4586]  (4586, 8094] (8094, 12140]
##             2             8             1             2             1
##  (12140, Inf]
##             1

## gr_gaps
##  [-Inf, 2536]  (2536, 4047]  (4047, 4586]  (4586, 8094] (8094, 12140]
##             2             8             1             2             1
##  (12140, Inf]
##             1
```

References

[1]Husereau, D., Drummond, M., Augustovski, F., de Bekker-Grob, E., Briggs, A. H., Carswell, C., Caulley, L., Chaiyakunapruk, N., Greenberg, D., Loder, E., Mauskopf, J., Mullins, C. D., Petrou, S., Pwu, R. F., Staniszewska, S., & CHEERS 2022 ISPOR Good Research Practices Task Force (2022). Consolidated Health Economic Evaluation Reporting Standards 2022 (CHEERS 2022) Statement: Updated Reporting Guidance for Health Economic Evaluations. Value in health : the journal of the International Society for Pharmacoeconomics and Outcomes Research, 25(1), 3–9. https://doi.org/10.1016/j.jval.2021.11.1351

[2]Krijkamp, E. M., Alarid-Escudero, F., Enns, E. A., Jalal, H. J., Hunink, M. G. M., & Pechlivanoglou, P. (2018). Microsimulation Modeling for Health Decision Sciences Using R: A Tutorial. Medical decision making : an international journal of the Society for Medical Decision Making, 38(3), 400–422. https://doi.org/10.1177/0272989X18754513

3Papaspyridakos, P., Chen, C. J., Singh, M., Weber, H. P., & Gallucci, G. O. (2012). Success criteria in implant dentistry: a systematic review. Journal of dental research, 91(3), 242â€“248. https://doi.org/10.1177/0022034511431252

4Sendi P, Bertschinger N, Brand C, Marinello CP, Bucher HC, Bornstein MM. Measuring the Monetary Value of Dental Implants for Denture Retention: A Willingness to Pay Approach. Open Dent J. 2017;11:498-502. Published 2017 Sep 14. doi:10.2174/1874210601711010498
