# Supplementary material for: Cost Effectiveness of Two Short Implants Versus One Short Implant With a Cantilever in the Posterior Region: 7.5‐Year Follow‐Up of a Randomised Controlled Trial
Source: J Clin Periodontol. 2025 Sep 21;52(12):1712–24. doi: 10.1111/jcpe.70039 (PMC12605678; doi:10.1111/jcpe.70039)
Supplement: Supplementary file 1 — Data S1: Supporting Information. [file JCPE-52-1712-s001.zip › jcpe70039-sup-0003-FigureS1@Supplement figures R1.docx]

**Supplement figures**

**
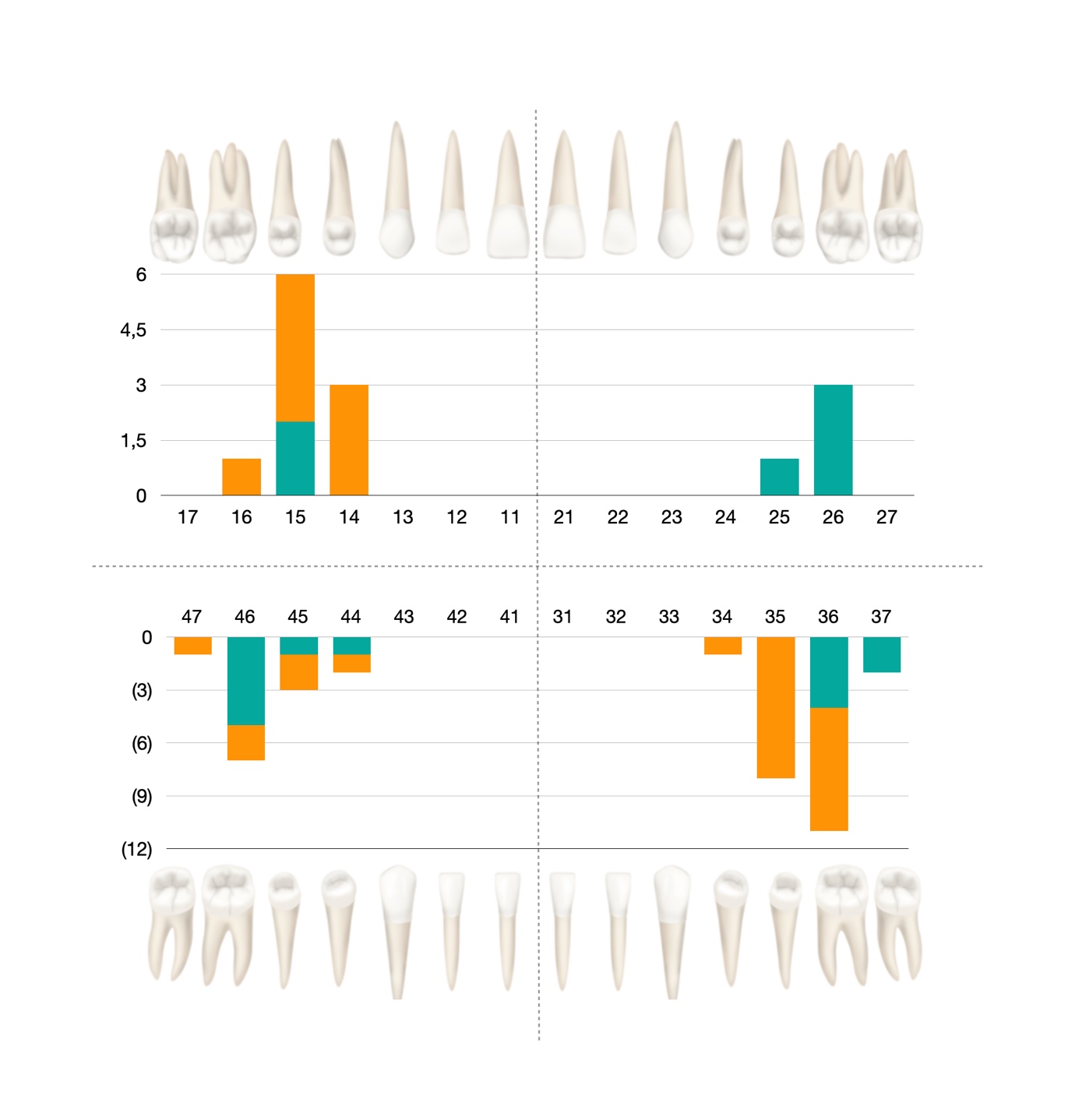
Supplement Figure 1.** Implant distribution and location

**
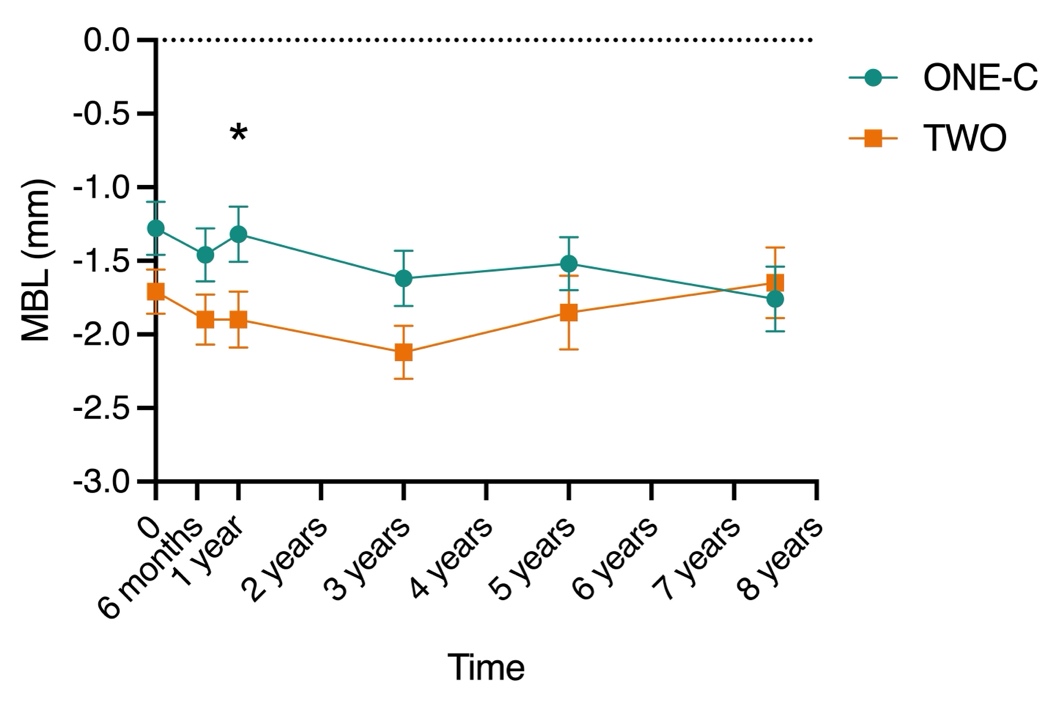
**

**Figure 4.** Line plot showing marginal bone levels over time in both treatment groups. Error bars indicate standard error. *p < .05 tested with a linear mixed model.
